# A Latex Metabolite Benefits Plant Fitness under Root Herbivore Attack

**DOI:** 10.1371/journal.pbio.1002332

**Published:** 2016-01-05

**Authors:** Meret Huber, Janina Epping, Christian Schulze Gronover, Julia Fricke, Zohra Aziz, Théo Brillatz, Michael Swyers, Tobias G. Köllner, Heiko Vogel, Almuth Hammerbacher, Daniella Triebwasser-Freese, Christelle A. M. Robert, Koen Verhoeven, Veronica Preite, Jonathan Gershenzon, Matthias Erb

**Affiliations:** 1 Root Herbivore Interactions Group, Max-Planck Institute for Chemical Ecology, Jena, Germany; 2 Department of Biochemistry, Max-Planck Institute for Chemical Ecology, Jena, Germany; 3 Fraunhofer Institute for Molecular Biology and Applied Ecology, Münster, Germany; 4 Institute of Plant Sciences, University of Bern, Bern, Switzerland; 5 Department of Entomology, Max-Planck Institute for Chemical Ecology, Jena, Germany; 6 Netherlands Institute of Ecology, Wageningen, Netherlands; The Sainsbury Laboratory, UNITED KINGDOM

## Abstract

Plants produce large amounts of secondary metabolites in their shoots and roots and store them in specialized secretory structures. Although secondary metabolites and their secretory structures are commonly assumed to have a defensive function, evidence that they benefit plant fitness under herbivore attack is scarce, especially below ground. Here, we tested whether latex secondary metabolites produced by the common dandelion (*Taraxacum officinale* agg.) decrease the performance of its major native insect root herbivore, the larvae of the common cockchafer (*Melolontha melolontha*), and benefit plant vegetative and reproductive fitness under *M*. *melolontha* attack. Across 17 *T*. *officinale* genotypes screened by gas and liquid chromatography, latex concentrations of the sesquiterpene lactone taraxinic acid β-D-glucopyranosyl ester (TA-G) were negatively associated with *M*. *melolontha* larval growth. Adding purified TA-G to artificial diet at ecologically relevant concentrations reduced larval feeding. Silencing the germacrene A synthase ToGAS1, an enzyme that was identified to catalyze the first committed step of TA-G biosynthesis, resulted in a 90% reduction of TA-G levels and a pronounced increase in *M*. *melolontha* feeding. Transgenic, TA-G-deficient lines were preferred by *M*. *melolontha* and suffered three times more root biomass reduction than control lines. In a common garden experiment involving over 2,000 *T*. *officinale* individuals belonging to 17 different genotypes, high TA-G concentrations were associated with the maintenance of high vegetative and reproductive fitness under *M*. *melolontha* attack. Taken together, our study demonstrates that a latex secondary metabolite benefits plants under herbivore attack, a result that provides a mechanistic framework for root herbivore driven natural selection and evolution of plant defenses below ground.

## Abstract 2

Dandelion plants protect their roots from the larva of the common cockchafer beetle by accumulating and releasing a sesquiterpene lactone deterrent in their exuded latex.

## Author Summary

Plant roots produce diverse and abundant blends of bioactive metabolites. One potential function of these compounds is to protect roots against the devastating effects of below ground herbivore attack. However, examples demonstrating such a protective function in native plant-herbivore systems are lacking. Here, we investigated the interaction between the dandelion (*T*. *officinale*) and its native root feeding enemy, larvae of the common cockchafer beetle (*M*. *melolontha*). Dandelion is known to release secondary metabolite-rich latex from wounded roots, thus we specifically focused on the potential defensive role of these metabolites. By combining natural variation, genetic manipulation and in vitro assays, we demonstrate that taraxinic acid glucoside, a highly concentrated chemical, deters cockchafer larvae and thereby protects the roots. Dandelion plants with high levels of taraxinic acid benefited from this protection in terms of both vegetative and reproductive fitness. Our study demonstrates that a latex metabolite benefits plant fitness under root herbivore attack, a result which provides a mechanistic framework for root herbivore driven natural selection and the evolution of root defenses.

## Introduction

Plants produce over 200,000 different metabolites that are not directly needed for their growth and development [1]. Many of these so-called secondary metabolites have a negative impact on insect herbivores [2–6], leading to the hypothesis that they evolved as defenses against the latter [7]. Indeed, recent studies demonstrated that leaf secondary metabolites reduce herbivore damage and thereby counteract the negative impact of herbivores on plant growth, that herbivore abundance covaries with secondary metabolites across different environments, that the exclusion of herbivores leads to rapid changes in genotype frequencies and associated metabolites, and that genes encoding for defensive metabolites can be under differential selection [8–12]. Together, these studies provide strong evidence for the hypothesis that above ground herbivores drive the evolution of leaf secondary metabolites.

In contrast to the leaves, less is known about the role of secondary metabolites in root–herbivore interactions. Roots are often attacked by below ground herbivores, and root herbivore infestation can strongly reduce plant growth and reproduction [13–15]. Furthermore, roots produce diverse and abundant blends of secondary metabolites [16,17], many of which can affect root herbivore behavior and reduce their performance [18]. Furthermore, root secondary metabolites can determine host species ranges in below ground feeding insects [19]. However, if root secondary metabolites enable plants to maintain growth (i.e., vegetative fitness) and reproduction (i.e., reproductive fitness) under root herbivore attack remains unclear. Common milkweed (*Asclepias syriaca*) families with high and low root cardenolides, for instance, did not differ in their above-ground biomass accumulation when attacked by *Tetraopes* *tetraophthalmus* below-ground [20]. Maize lines with high root benzoxazinoid concentrations on the other hand suffered less root damage by *Diabrotica virgifera virgifera* and had higher yields than lines with low benzoxazinoid concentrations [21]. However, follow-up experiments conducted under more controlled conditions failed to confirm this pattern [5,22]. The lack of knowledge regarding fitness benefits of root secondary metabolites makes it difficult to understand their role in the evolution of plant–herbivore interactions.

In both leaves and roots, secondary metabolites often accumulate in specialized structures including laticifers [23,24], which are among the most common secretory structures of flowering plants [25–27]. Laticifers are elongated individual or interconnected cells whose cytoplasm is called latex [28,29]. Laticifers are often under pressure and release large quantities of latex upon wounding, which can deter or even kill insect herbivores [28,30]. Surprisingly, however, direct evidence that laticifers are defensive, i.e., that they are positively associated plant vegetative or reproductive fitness in the presence but not in the absence of herbivory, is virtually absent [28,31,32]. A study by Agrawal [31] showed that latex exudation is under positive selection in common milkweed under ambient insect pressure. However, whether this pattern is herbivore dependent remains to be elucidated.

One of Europe’s most prevalent native latex-producing plants is the common dandelion (*T*. *officinale* agg.) (Flora Helvetica, 5th edition). *T*. *officinale* is a species complex consisting of sexual, outcrossing diploids that are native to central and southern Europe and a multitude of apomictic, clonal triploids that are spreading across the globe [33–35]. Similar to many other perennials in temperate ecosystems, the plant relies on its roots for resprouting and flowering in spring. As a perennial plant, both vegetative and reproductive performance contribute to the fitness of the plant. *T*. *officinale* produces latex in all major organs, with the highest amounts exuding from wounded tap roots [36]. The latex is dominated by three classes of secondary metabolites: phenolic inositol esters (PIEs), triterpene acetates (TritAcs) and the sesquiterpene lactone taraxinic acid β-D-glucopyranosyl ester (TA-G) [36]. Each compound class accounts for 5%–7% of latex fresh mass [36]. Sesquiterpene lactones and TritAcs can have deterrent and toxic effects against a wide range of organisms [37–40]. In its native range, *T*. *officinale* is frequently attacked by the larva of the common cockchafer (also called May bug), *M*. *melolontha* (Coleoptera: Scarabaeidae). *M*. *melolontha* is among Europe’s largest and most prevalent native root-feeding insects and periodically causes devastating damage to crops and pastures [41–43]. Although the larvae are highly polyphagous, they preferentially feed on *T*. *officinale* [44,45].

In this study, we explored the putative defensive function of *T*. *officinale* latex secondary metabolites against *M*. *melolontha* larvae. First, we investigated which latex secondary metabolites are likely to be involved in root herbivore defense using a correlative approach. Second, we decreased the production of the major candidate compound TA-G by identifying the gene encoding the first committed biosynthetic step and silencing it by RNA interference (RNAi), which allowed testing the effect of TA-G deficiency on plant and insect performance. Third, we purified TA-G to investigate its impact on *M*. *melolontha* in vitro. Fourth, we performed a common garden experiment with different *T*. *officinale* genotypes to determine whether TA-G reduces the negative impact of *M*. *melolontha* on plant vegetative and reproductive performance in the field. Through the above approaches, we demonstrate that TA-G protects the roots and thereby benefits plant fitness in the presence of root herbivores.

## Results

### The Concentration of the Constitutively Produced Sesquiterpene Lactone TA-G in the Latex Is Negatively Correlated with *M*. *melolontha* Performance

**Fig 1:**
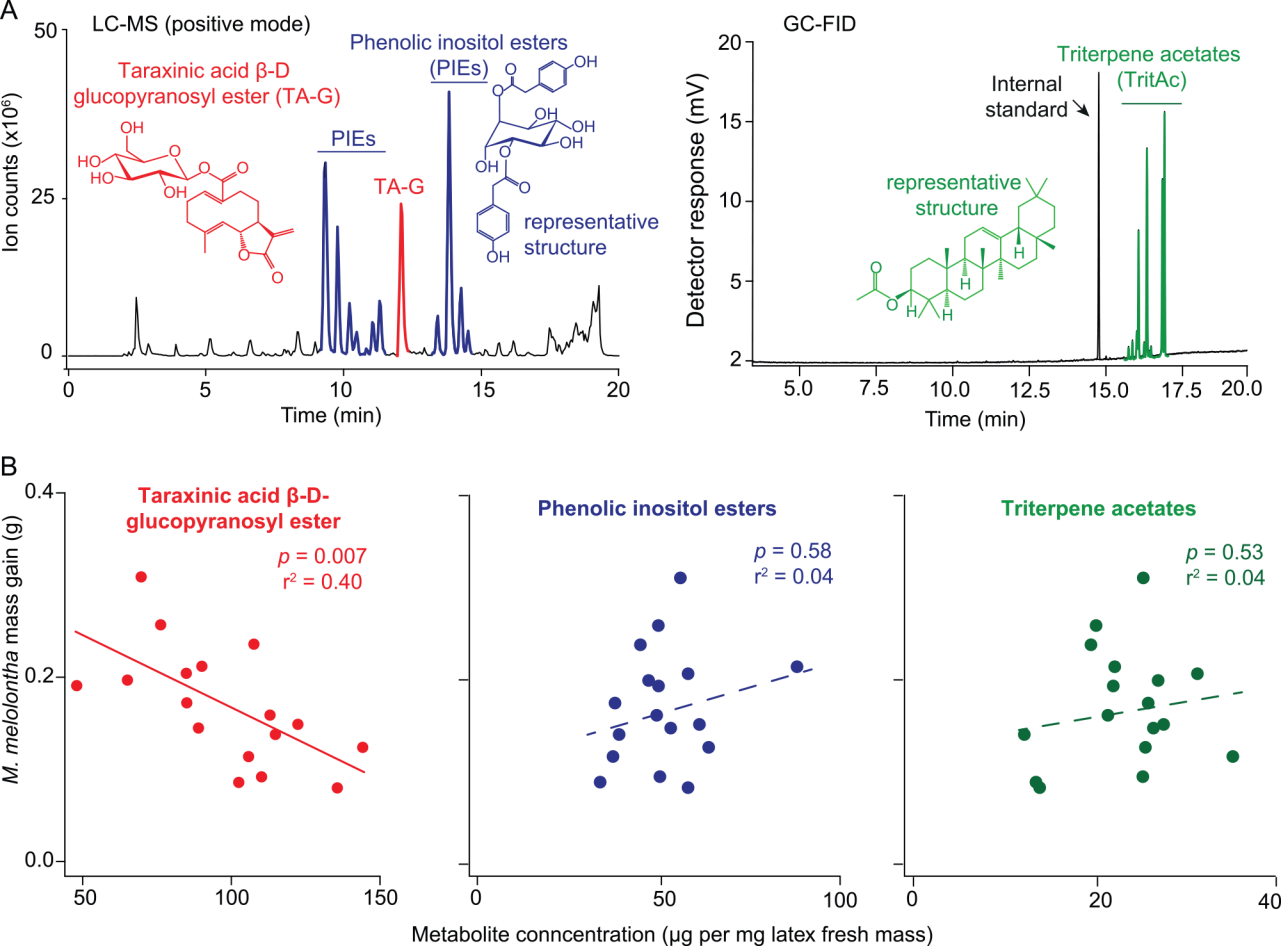
*M*. *melolontha* growth correlates negatively with the concentration of the latex metabolite TA-G. A. Representative liquid chromatography-mass spectrometry (LC-MS) chromatogram of a latex methanol extract (left panel) and gas chromatography-flame ionization detector (GC-FID) chromatogram of a latex hexane extract (right panel) depicting the three major classes of latex secondary metabolites in *T*. *officinale*. LC = liquid chromatograph. MS = mass spectrometer. GC = gas chromatograph. FID = flame ionization detector. B. After 11 d of feeding, growth of *M*. *melolontha* larvae on 17 *T*. *officinale* genotypes was negatively correlated with TA-G concentration in the root latex (linear model, *p* = 0.007, left panel). *M*. *melolontha* growth was not correlated with the total concentrations of PIEs (middle panel) or TritAcs (right panel). Each data point represents the mean *M*. *melolontha* growth rate of 12 independent replicates of one *T*. *officinale* genotype. Underlying data can be found in S1 Data.

Three classes of secondary metabolites dominate the latex of *T*. *officinale*: PIEs (Fig 1A, left panel), the sesquiterpene lactone TA-G (Fig 1A, left panel), and TritAcs (Fig 1A, right panel) [36]. We measured the concentrations of the major latex secondary metabolites in 40 triploid *T*. *officinale* genotypes collected across central and northern Europe and selected 17 genotypes that displayed maximal variation in latex traits, but minimal variation in growth (S1 Text, S1 Table) to correlate latex secondary metabolites with herbivore performance. *M*. *melolontha* larval mass gain was negatively correlated with the concentration of TA-G (Fig 1B, left panel, *p* = 0.007, r^2^ = 0.40, linear model), with TA-G accounting for 26% of the observed variance. By contrast, larval mass gain was not correlated to the total concentrations of PIEs or TritAcs (Fig 1B, middle and right panel, *p* = 0.58 for PIEs; *p* = 0.53 for TritAcs, *n* = 17, linear models). Also, the concentration of TA-G was not correlated to the amount of latex that was released from wounded roots (S1 Fig). Surprisingly, latex mass was positively correlated to larval mass gain when analyzed together with TA-G concentration using multiple linear regression (S2 Table, *p*(TA-G) = 0.003, *p*(latex mass) = 0.03, linear model). The total amount of TA-G (latex mass * TA-G concentration), on the other hand, was not correlated to larval mass gain (S3 Table). Across the different genotypes, TA-G was constitutively produced and not induced by *M*. *melolontha* attack. On the contrary, we observed a trend for a reduction of TA-G concentration in the latex of *M*. *melolontha*-attacked roots (S2 Fig, *p* = 0.08 *t*-test,). The magnitude of this effect was similar across genotypes (S3 Fig, *p* = 0.0004, linear model).

To test if TA-G predominantly accumulates in laticifers and to what extent this accumulation is responsible for the overall TA-G concentration in the roots, we measured TA-G concentrations in latex-drained and latex-containing main roots, as well as latex-free root cortex cells. Draining latex from the roots decreased TA-G concentration by a factor of four (S4 Fig, *p* = 4x10^-6^, one-way ANOVA). TA-G concentration in the root cortex was as low as in drained roots (S4 Fig, *p* = 4x10^-6^, one-way ANOVA). Across the 17 different genotypes, TA-G concentrations in the latex and in the entire main roots were strongly positively correlated, with TA-G concentrations being about 100-fold higher in latex than in main roots (S5 Fig, *p* = 0.004, linear model). Together, these experiments show that TA-G is predominantly stored in the laticifers, and that latex TA-G is responsible for the overall concentration of TA-G in *T*. *officinale* roots.

### Genetic Manipulation Shows That TA-G Deters *M*. *melolontha* and Reduces Loss of Root Biomass to Herbivory

**Fig 2:**
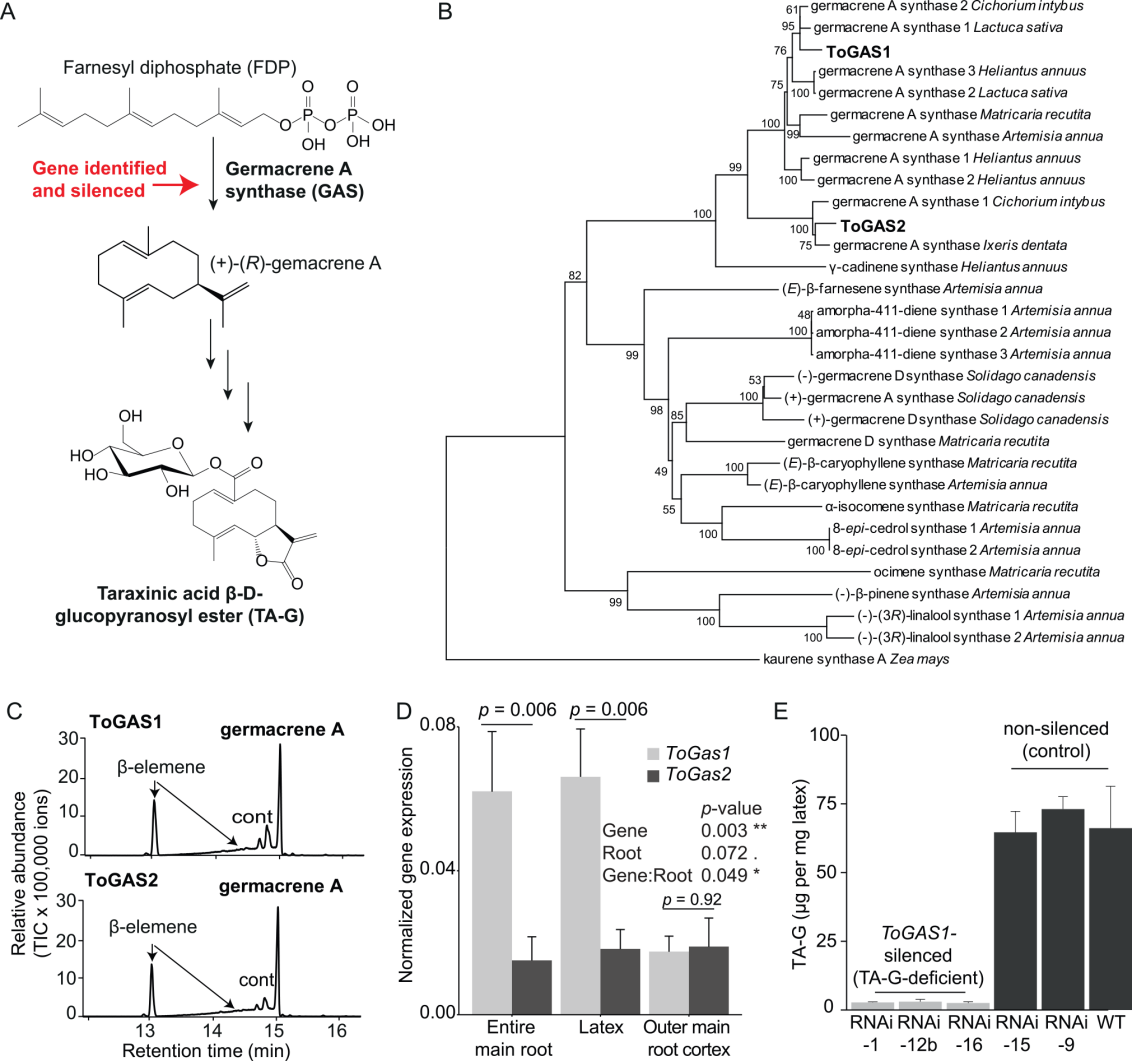
The germacrene A synthase ToGAS1 mediates the first committed step in latex TA-G biosynthesis. A. Partial biosynthetic pathway of TA-G. B. Phylogenetic tree of the newly identified *T*. *officinale* germacrene A synthases ToGAS1/2 and known Asteraceae terpene synthases (neighbor-joining method, *n* = 1,000 replicates). Bootstrap values are shown next to each node. Accession numbers can be found in S4 Table. C. GC-MS analysis of enzyme products from recombinant ToGAS1/2 expressed in *Escherichia coli* and incubated with the substrate FDP. Germacrene A is converted to β-elemene during hot GC injection. cont, contamination. GC-MS = gas chromatograph coupled to a mass spectrometer. D. Expression of *T*. *officinale* germacrene A synthase genes (*ToGAS1* and *ToGAS2*) in the entire main root, latex, and outer main root cortex as determined by RT-qPCR. Statistics of two-way ANOVA and pairwise comparison according to Tukey’s post hoc test are shown. Mean Sq = Mean of squares. *n* = 3. E. Silencing of *ToGAS1* by RNAi generated three independently silenced lines with strongly depleted TA-G concentrations and two transformed, nonsilenced lines with similar TA-G concentrations as the parental wild type. N = 3. Underlying data can be found in S1 Data.

To investigate the effect of TA-G on *M*. *melolontha* preference and *T*. *officinale* performance, we identified and silenced a gene that encodes for a germacrene A synthase, the enzyme that mediates the first committed step of TA-G biosynthesis, by RNAi (Fig 2A). To identify germacrene A candidate genes in *T*. *officinale*, we sequenced a transcriptome of the main root and the latex and constructed a reference transcriptome with the pooled reads. Putative germacrene A synthases were identified based on amino acid sequence similarity with two known germacrene A synthases from chicory [46]. Through this approach, we obtained full-length sequences of two putative germacrene A synthase genes, *ToGAS1* and *ToGAS2*, which share 71% identity at the amino acid level. Phylogenetic comparison with other Asteraceae terpene synthases revealed that ToGAS1 belongs to the larger of two germacrene A synthase clusters, while ToGAS2 belonged to the smaller cluster (Fig 2B). Heterologous expression in *Escherichia coli* showed that both recombinant proteins produced (+)-germacrene A when incubated with the substrate farnesyl diphosphate (FDP) (Fig 2C, S6 Fig). To further characterize the two genes, we analyzed their expression in the outer root cortex, latex, and the entire main root. As *ToGAS1* was more strongly expressed than *ToGAS2* in both latex and entire main roots (Fig 2D), we targeted *ToGAS1* through RNAi by expressing a 191 base pair fragment of this gene under the control of the constitutive *35S* promoter. A reduction of TA-G by over 90% compared to wild type was observed in three independently transformed lines: −1, −12b, and −16 (“TA-G-deficient lines”). No reduction in TA-G concentration was found in two other lines, −9 and −15, compared to wild type (all designated as “control lines”) (Fig 2E). The amount of exuded latex did not differ between TA-G-deficient and control lines (S7 Fig). *ToGAS1* was suppressed by more than 90% in the TA-G deficient lines compared to control lines, whereas *ToGAS2* expression was not affected (S8 Fig). These results show that ToGAS1 is involved in TA-G biosynthesis in *T*. *officinale* latex.

**Fig 3:**
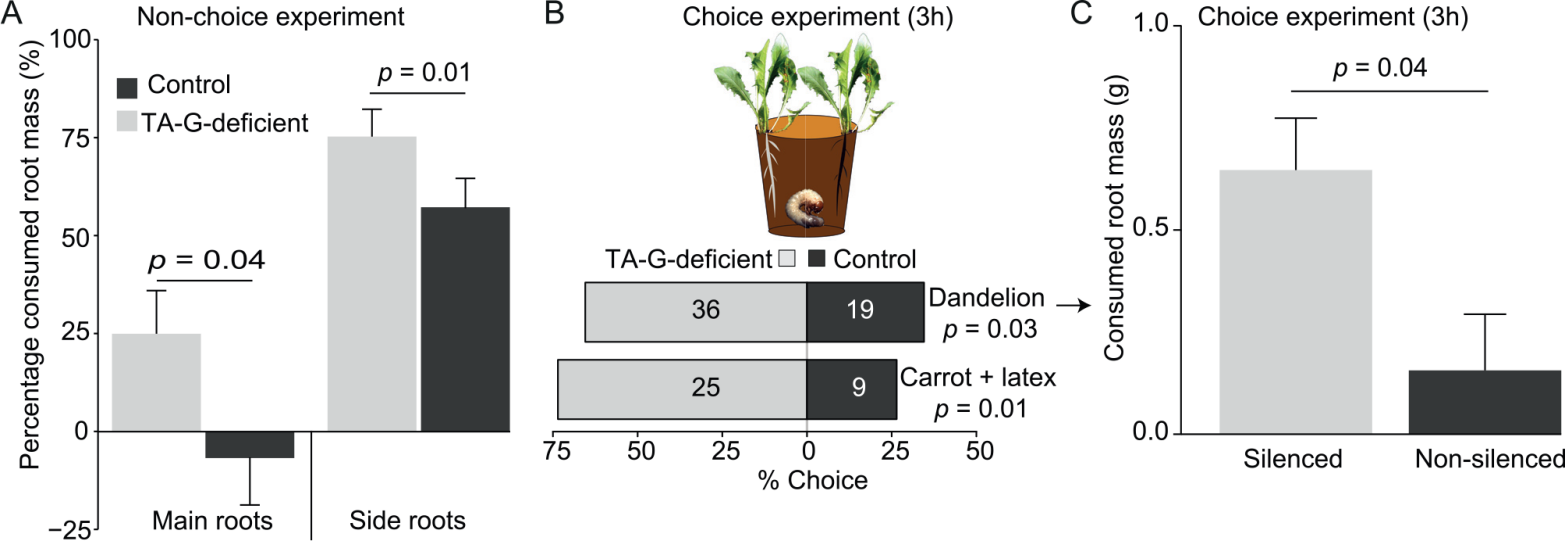
Silencing of the germacrene A synthase gene *ToGAS1* increases *M*. *melolontha* feeding. A. In a nonchoice experiment, TA-G-deficient lines lost more main and side root mass than control lines after 10 d of feeding by *M*. *melolontha* relative to undamaged control plants of each accession (“relative root mass”) (Kruskal-Wallis rank sum test, *n* = 36). B. TA-G-deficient *T*. *officinale* (top bar) and carrot seedlings painted with latex from TA-G-deficient *T*. *officinale* (lower bar) were preferred by *M*. *melolontha* over controls after three hours of feeding (binomial test). Diagrams show pooled data of all possible pairwise comparisons of individual TA-G-deficient and control lines. Numbers inside bars refer to number of larvae. C. *M*. *melolontha* consumed more root mass from TA-G-deficient *T*. *officinale* seedlings compared to control seedlings in a choice experiment after 4 h (paired Student’s *t* test, *n* = 81). Underlying data can be found in S1 Data.

To test the function of TA-G in planta using the transgenic lines, we first measured the effect of *M*. *melolontha* attack on 8 wk-old TA-G-deficient and control *T*. *officinale* lines. As noninfested TA-G-deficient and control lines differed in their growth (S9 Fig), we expressed the biomass of herbivore-infested plants relative to the mean biomass of control plants of each genotype. After herbivory, TA-G-deficient lines had lower main and side root mass (Fig 3A, main roots: *p* = 0.04; side roots: *p* = 0.01, Kruskal-Wallis rank sum test), but not leaf mass (S10 Fig, *p* = 0.8, Kruskal-Wallis rank sum test), expressed relative to noninfested plants of each genotype, showing that TA-G-deficient lines suffered a higher percentage of root biomass reduction than control lines. To exclude the possibility that the observed effects are due to differences in root growth, we performed a choice experiment with the TA-G-deficient and control lines using 5 wk-old plants, which did not show any differences in growth or biomass accumulation (S11 Fig). *M*. *melolontha* larvae preferred to feed on TA-G-deficient rather than on control lines (Fig 3B, top panel, *p* = 0.03, binomial test), resulting in three times higher root mass loss in the TA-G deficient than in the control lines under *M*. *melolontha* attack (Fig 3C, *p* = 0.04, paired Student’s *t* test). Additional metabolic profiling revealed that TA-G-deficient and control lines differed in total root protein levels (S12–S14 Figs). However, no correlation of this trait with *M*. *melolontha* behavior was found (S15 Fig). To specifically test the effect of TA-G silencing on latex bioactivity, we painted 6 wk-old carrot seedlings with latex from TA-G-deficient and control plants. *M*. *melolontha* preferred to feed on carrots painted with latex from the TA-G-deficient lines compared to that from the control lines as measured three hours after the start of the experiment (Fig 3B, lower panel, *p* = 0.01, binomial test).

### Addition of Purified TA-G Deters *M*. *melolontha* Feeding

**Fig 4:**
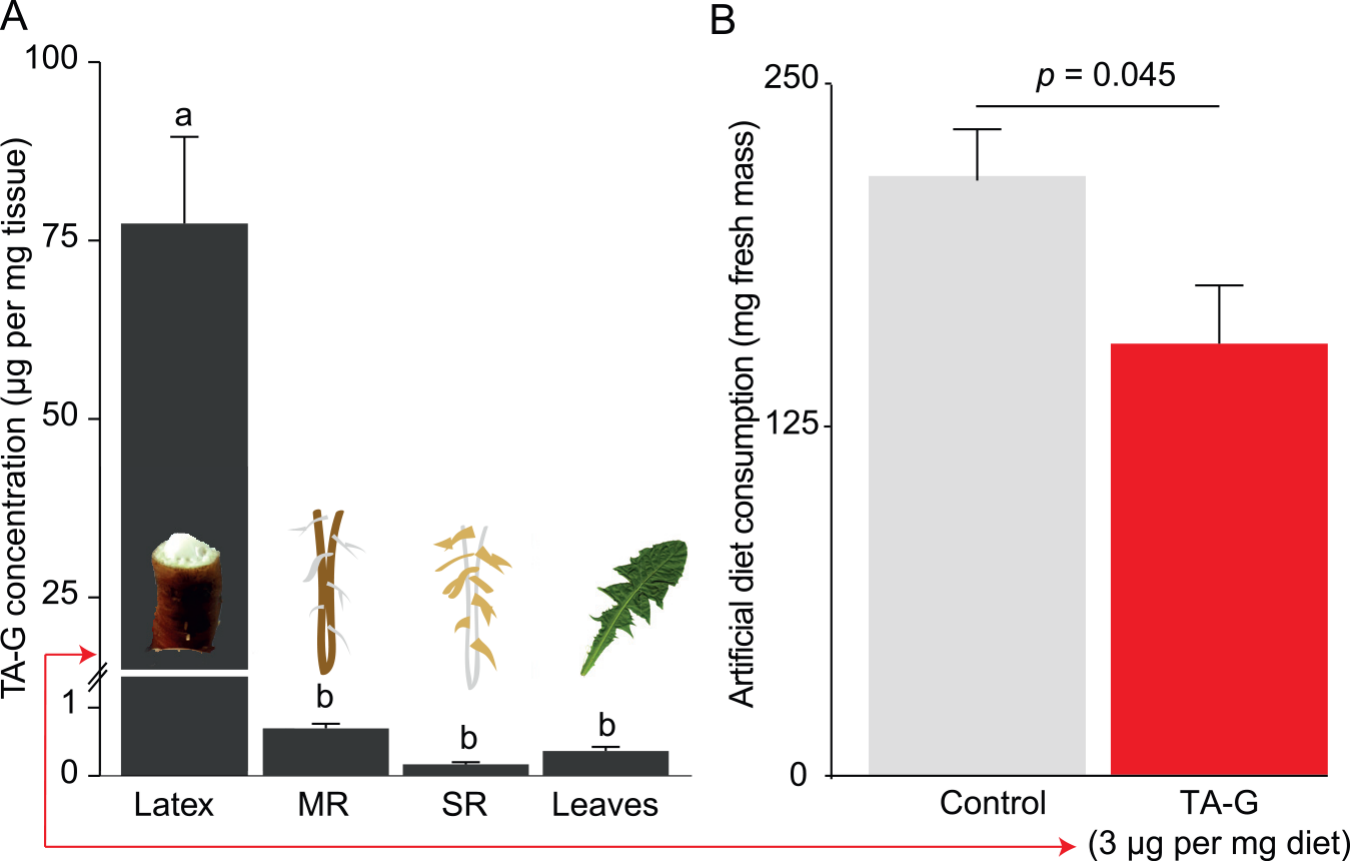
TA-G reduces larval feeding on artificial diet at ecologically relevant concentrations. A. TA-G concentration across tissues. B. *M*. *melolontha* consumed 40% less TA-G-containing diet compared to control diet in a nonchoice experiment after 24 h (Student’s *t* test, *n* = 15). Underlying data can be found in S1 Data.

Latex profiling revealed that TA-G-deficient lines also had lower PIE levels, suggesting an interaction between the two pathways (S16 and S17 Figs). To test whether TA-G alone is sufficient to reduce larval consumption, we isolated and purified TA-G by preparative chromatography and performed a feeding experiment with *M*. *melolontha* larvae feeding on artificial diet containing TA-G. To determine physiologically relevant TA-G concentrations, we first quantified TA-G in different *T*. *officinale* tissues. Latex contained 75 μg TA-G per mg per fresh mass, and the main roots, side roots and leaves contained 0.2–0.7 μg TA-G per mg fresh mass (Fig 4A). For the artificial diet experiment, we used a concentration of 3 μg TA-G per mg diet to represent a natural situation in which *M*. *melolontha* feeds on a root that accumulates latex at the site of wounding. Over 24 h, *M*. *melolontha* larvae consumed 40% less TA-G containing diet than control diet (Fig 4B, *p* = 0.045, Student’s *t* test).

### TA-G Reduces the Negative Impact of *M*. *melolontha* Herbivory on Plant Vegetative and Reproductive Fitness under Field Conditions

**Fig 5:**
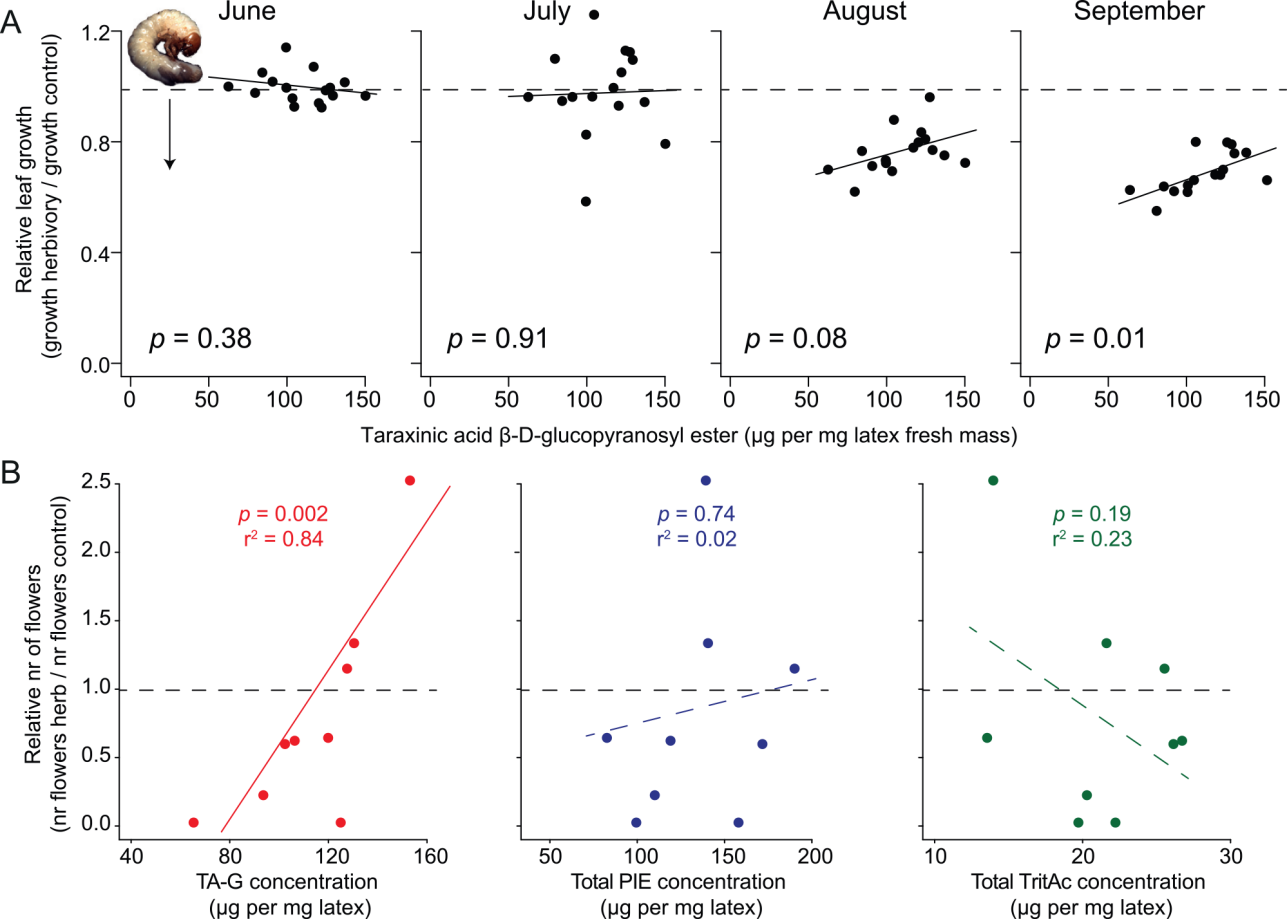
TA-G reduces the negative effect of *M*. *melolontha* on plant vegetative and reproductive performance in the field. A. TA-G concentration was positively correlated to relative leaf growth across 17 *T*. *officinale* genotypes in a common garden experiment towards the end of the growing season. Relative leaf growth is the mean leaf growth of herbivore-infested plants of each genotype during the infestation period compared to the mean leaf growth of the control plants of each genotype (leaf growth: increase in maximal leaf length compared to maximal leaf length before infestation). Data points below the horizontal dashed line indicate reduction in leaf growth under *M*. *melolontha* attack. Each data point represents the mean of one genotype. Plants were infested at the end of June. Statistics of Pearson’s product-moment correlations based on mean values per genotype are shown. B. The relative number of flowers (number of flowers of the herbivore-infested plants expressed relative to noninfested plants of each genotype) was positively correlated with the concentration of TA-G, but not with the total concentrations of PIEs or TritAcs at the beginning of the flowering season. Only genotypes that flowered at this time point are shown (9 out of 17 genotypes). Underlying data can be found in S1 Data.

To investigate whether TA-G benefits vegetative and reproductive fitness under *M*. *melolontha* attack in the field, we grew 2,040 *T*. *officinale* individuals of the experimental population (consisting of the 17 genotypes as described above) in a common garden. We established 20 circular plots, each of them containing 6 individuals of each genotype, and infested half of the plots with 72 *M*. *melolontha* larvae (23 larvae per m^2^) each (S18 Fig), a density similar to the damage threshold in pastures [47]. In the first year during which most plants did not flower, we measured the length of the longest leaf (“maximal leaf length”)—a reliable predictor for leaf and root mass under greenhouse conditions (S19 Fig)—and correlated this parameter with latex secondary metabolite concentrations. To standardize growth rates, we expressed the size increase of the longest leaf of the herbivore-infested plants relative to the size increase of the longest leaf of control plants of the same genotype (“relative leaf growth”). Shortly after infestation of the plants in June, no reduction in leaf growth was observed in the infested plants, and relative leaf growth was not correlated with the concentration of the three latex secondary metabolite classes (Fig 5A, *p*(June) = 0.38, Pearson’s product–moment correlation). In the course of the growing season, *M*. *melolontha* infestation reduced overall plant growth, and a positive correlation between relative leaf growth and TA-G concentration emerged, suggesting that TA-G reduced the negative impact of *M*. *melolontha* on plant performance (Fig 5A, *p*(September) = 0.01, Pearson’s product–moment correlation). In absolute terms, TA-G concentration and leaf growth tended to be positively correlated under *M*. *melolontha* attack and negatively correlated in the absence of *M*. *melolontha* (S20 Fig). No correlation between relative leaf growth and the total concentrations of PIEs, TritAcs, latex mass, or the total amount of TA-G (latex mass * TA-G concentration) was observed throughout the entire growing season (S21 Fig, S5 Table). Similarly, latex mass did not significantly account for relative leaf length when analyzed in a multiple regression together with TA-G concentration (S6 Table). Leaf length of the herbivore-infested plants was proportional to leaf length of noninfested plants, indicating that plant size did not affect the degree of damage (S22 Fig). To assess whether TA-G also benefits plant reproductive fitness, we correlated the number of flowers to latex secondary metabolite concentration in the following year. At the beginning of the flowering season, TA-G was positively correlated with the relative number of flowers (number of flowers of the herbivore-infested plants expressed relative to noninfested plants of each genotype) in the genotypes that flowered at this time point (Fig 5B, left panel). Genotypes that flowered did not differ in their TA-G concentration from genotypes that did not flower at this time point (*p* = 1, Wilcoxon rank sum test). No correlation between the relative number of flowers and the total concentrations of PIEs and TritAcs were observed (Fig 5B, middle and right panel). The positive correlation between the relative number of flowers, and TA-G disappeared at the end of the flowering period (*p* = 0.33, Pearson’s product–moment correlation), likely because almost all *M*. *melolontha* larvae had stopped feeding by this time (S7 Table). Together, these data strongly suggest that TA-G reduces the negative effect of root herbivore attack on plant vegetative and reproductive fitness.

## Discussion

In this study, we demonstrate that the sesquiterpene lactone TA-G, a major secondary metabolite of *T*. *officinale*, protects the plant against its major native root herbivore *M*. *melolontha*. TA-G deters *M*. *melolontha* larvae from feeding and thereby directly protects the roots, resulting in a reduction of the negative impact of the root feeder on vegetative and reproductive fitness. The observed pattern indicates that root herbivores may exert positive selection pressure on latex secondary metabolites and may thereby drive their evolution.

Our experiments involving natural variation, chemical manipulation, and genetic modification provide parallel lines of evidence for a negative effect of TA-G on *M*. *melolontha* larvae. First, across different *T*. *officinale* genotypes, TA-G concentration was negatively correlated with *M*. *melolontha* growth. Surprisingly, latex mass was positively associated with larval mass gain. Other unidentified plant traits that benefit the larvae and covary with latex exudation may account for this pattern. Second, purified TA-G reduced food consumption in vitro. Third, TA-G suppression through *ToGAS1*-silencing increased the attractiveness and consumption of *T*. *officinale* roots and decreased the deterrent effect of *T*. *officinale* latex towards *M*. *melolontha*. Interestingly, silencing *ToGAS1* not only affected sesquiterpene lactone biosynthesis, but also plant growth and the accumulation of PIEs. Germacrene A synthases convert FDP into germacrene A. The substrate FDP is a common precursor for sesquiterpenes, triterpenes, and phytosterols [48,49], and the farnesyl residue can bind to growth-regulating proteins of the *ras* family [50]. It is therefore possible that *ToGAS1* silencing affects other branches of the metabolism of *T*. *officinale* by changing FDP pool sizes. These observations illustrate the limitations of transgenic approaches as a stand-alone method and highlight the power of combining genetic manipulation, natural variation, and chemical complementation to elucidate the role of plant secondary metabolites in plant–herbivore interactions.

Many studies demonstrate that plant secondary metabolites are toxic to root and leaf herbivores [2–6]. Surprisingly, however, the benefits for the plant often remain unclear, especially for root herbivores [28,51,52]. Plant secondary metabolites may reduce food quality for herbivores and may thereby trigger compensatory feeding, leading to higher plant damage [53]. In addition, herbivore-imposed loss of biomass can lead to an overcompensation of plant growth and sexual reproduction, which may mask the fitness benefits of resistance factors [54–56]. Secondary metabolites can also reduce plant performance in the field by attracting specialized herbivores that use the chemicals as oviposition [57] and foraging cues [22,58,59]. All these factors may constrain the fitness benefits of bioactive secondary metabolites. Finally, the heterogeneity of natural environments, including varying herbivore communities and abiotic factors, can render the detection of fitness benefits difficult [10–12]. We manipulated the abundance of a major root herbivore within artificial populations consisting of plant genotypes that differ substantially in their capacity to produce plant secondary metabolites. This approach allowed us to demonstrate herbivore-dependent vegetative and reproductive fitness benefits under field conditions. Similar experimental designs could be used in combination with transgenic or genetic mapping populations to quantify the contribution of individual herbivore species and herbivore communities to secondary metabolite-dependent fitness benefits in heterogeneous environments [10–12].

So far, clear evidence for the fitness advantage of particular metabolites under insect attack has remained particularly scarce for below-ground plant–herbivore interactions. Vaughan et al. [60] showed that silencing the production of a semivolatile diterpene increased root damage of *Arabidopsis thaliana* by the opportunistic fungus gnat *Bradysia* spp. However, it remains unknown to what extent this effect translates into improved plant performance in nature. The lack of knowledge regarding the benefits of secondary metabolites under root herbivory limits our understanding for the evolution of root metabolites. *Eschscholzia californica* (Fabaceae) mainland populations that are exposed to pocket gopher herbivory had 2.5 times higher root alkaloid concentrations than island populations that are free from this herbivore pressure [61], suggesting that pocket gophers may exert positive selection on this metabolite. We show here that high TA-G concentration benefits plant vegetative and reproductive performance in the presence of *T*. *officinale*’s major native root herbivore, *M*. *melolontha*, thus providing an evolutionary framework for root herbivore-driven natural selection. TA-G-deficient *T*. *officinale* lost more root mass than control lines upon feeding by *M*. *melolontha*. In a common garden experiment, TA-G concentration was positively correlated with leaf growth and flower production across natural *T*. *officinale* genotypes.

While our data provides evidence that TA-G benefits the plants under *M*. *melolontha* attack, we did not obtain strong evidence for costs of TA-G production. Although TA-G concentration tended to be negatively correlated to plant growth across the 17 genotypes in the common garden experiment in the absence of *M*. *melolontha*, the correlation was not significant, possibly due to the relatively low number of genotypes that were used for the experiment. Experiments that evaluate putative fitness costs of TA-G production in different environments may provide further insights into the varying fitness effects of TA-G.

Laticifers are commonly assumed to be defensive [28,30]. Toxic metabolites or proteins in the latex can reduce herbivore performance [28], while the stickiness of latex can trap entire insects or glue their mouthparts together [30,62]. Despite the overwhelming evidence that latex reduces herbivore performance, experimental validation that latex benefits plant fitness under herbivore attack remains scarce [31,32]. We show that a toxic metabolite in the latex benefits the plant in the presence but not in the absence of an herbivore and thereby provide an experimental validation of the assumption that microevolutionary processes govern intraspecific variation in plant defense traits. These microevolutionary processes are consistent with the observed macroevolutionary patterns in which latex represents a key innovation that has spurred the evolution of the angiosperms [25]. Taken together, our results furnish an ecological and evolutionary explanation for the high concentrations of root and latex secondary metabolites and highlight the potential of soil-dwelling insects to shape the chemical defenses of their host plants.

## Materials and Methods

### Plant Growth Conditions

All indoor experiments were performed in a climate chamber operating under the following conditions: 16 h light 8 h dark; light supplied by a sodium lamp NH 360 FLX SUNLUX ACE Japan; light intensity at plant height: 58 μmol m^2^ s^−1^; temperature: day 22°C; night 20°C; humidity: day 55%, night 65% (unless specified otherwise). Plants were potted in 0.7–1.2 mm sand and watered with 0.01%–0.05% fertilizer with N-P-K of 15-10-15 (Ferty 3, Raselina, Czech Republic).

### Insects

*M*. *melolontha* larvae (S23 Fig) were collected from meadows in Switzerland and Germany. Experiments were performed with larvae in the third larval stage (L3) unless indicated otherwise. Insects were reared individually in 200 ml plastic beakers filled with a mix of potting soil and grated carrots in a phytotron operating under the following conditions: 12 h day 12 h night; temperature: day 13°C, night 11°C; humidity: 70%; lighting: none.

### Statistical Analyses

All statistical analyses were performed in R version 3.1.1 [63]. Pairwise comparisons were performed with the agricolae [64] and lsmeans [65] package. Results were displayed using ggplot2 [66] and gridExtra [67]. More details on the individual statistical procedures are given in the experimental sections below.

### Correlations between Latex Secondary Metabolites and *M*. *melolontha* Performance

To investigate the effects of latex secondary metabolites on *M*. *melolontha* performance, we measured growth of *M*. *melolontha* larvae on 17 *T*. *officinale* genotypes. To establish an experimental *T*. *officinale* population, we screened 40 triploid genotypes from central and northern Europe [68] for secondary metabolite concentrations and growth rates. Twenty genotypes were selected based on maximal difference of latex chemistry with minimal variation in plant growth rate using cluster analysis (S1 Table, S1 Text). Among these 20 genotypes, three genotypes completely lacked TA-G but were later found to contain other unidentified sesquiterpene lactone glycosides (S24 Fig). These genotypes were subsequently excluded from analysis. The remaining 17 genotypes were used to correlate larval growth with latex secondary metabolite concentrations. For each genotype, 12 plants were infested at an age of 7 wk with one preweighed *M*. *melolontha* larva, while 12 plants were left herbivore-free. Eleven days after infestation, *M*. *melolontha* larvae were recovered and larval mass difference was determined. To measure the concentration of latex secondary metabolites, main roots were cut 1 cm below the tiller and exuding latex collected into Eppendorf tubes and glass vials, immediately flash-frozen in liquid nitrogen and stored at −80°C until extraction.

For extraction, 1 ml methanol was added to the plastic tubes, and 1 ml hexane containing 0.1 mg*ml^−1^ cholesteryl acetate as internal standard was added to the glass vials. Both types of vessels were vortexed for 5 min, centrifuged, and the supernatant was stored at −80°C until analysis. Methanol samples were measured on a high pressure liquid chromatograph (HPLC 1100 series equipment, Agilent Technologies), coupled to a photodiode array detector (G1315A DAD, Agilent Technologies) and an Esquire 6000 ESI-Ion Trap mass spectrometer (Bruker Daltonics, Bremen, Germany). For quantification, peak areas were integrated at 245 nm for TA-G and at 275 nm for PIEs, and quantified using external standard curves. Hexane samples were analyzed on an Agilent series 6890 gas chromatograph coupled to a flame ionization detector (GC-FID). Individual TritAcs were quantified based on the internal standard. Methodological details for the analytical procedure have previously been described [36].

Correlations between TA-G, total PIE, total TritAc concentrations and total amount of TA-G (TA-G concentration * latex mass) and *M*. *melolontha* mass gain, as well as between TA-G concentration and latex fresh mass, were analyzed using linear models on the mean values of each of the 17 genotypes using the metabolite concentration and latex mass of the noninfested plants, as these measurements were not confounded by differential larval feeding activities. The combined effect of TA-G concentration and latex fresh mass on *M*. *melolontha* growth was analyzed with a multiple regression based on the mean values of the 17 genotypes of the control treatment. Differences in TA-G concentration between *M*. *melolontha*-infested and control plants were analyzed with Student’s *t* tests. The correlation across the 17 genotypes between TA-G concentrations of the control, and *M*. *melolontha*-infested plants were analyzed with a linear model. The assays were performed in two blocks within two months, and latex for GC analysis was collected from a third batch of plants grown in the same growth chamber under identical conditions.

### Correlations between TA-G Concentrations in the Latex and Main Roots

To investigate to which extent latex contributes to TA-G measured in the main roots, we measured the correlation between TA-G concentration in the latex and main roots, as well as the difference in TA-G concentration between main roots, latex-drained main roots, and largely latex-free outer main root cortex. To analyze the correlation of TA-G concentration in the latex and main roots, we grew 12 plants of each of the above-mentioned 17 genotypes. Main roots of 9 wk-old plants were cut 1 cm below the tiller and the exuding latex was collected. Main roots were separated from side roots and flash-frozen in liquid nitrogen. Latex was extracted using 1 ml methanol containing 10 μg*ml^−1^ loganin as an internal standard and analyzed on HPLC-DAD as described above. Peak area was integrated at 245 nm for TA-G and normalized to loganin as an internal standard. Main roots were ground to a fine powder, and 100 mg tissue was extracted with 1 ml methanol containing 10 μg*ml^−1^ loganin, vortexed, centrifuged, and the supernatant transferred to HPLC vials. Main root samples were analyzed the same way as the latex samples, except that the mobile phases consisted of 0.1% acetic acid (A) and acetonitrile (B) using following gradient: 0 min: 5% B, 18 min: 43% B, followed by column reconditioning. Peak area was integrated at 245 nm for TA-G and normalized to loganin as internal standard. The correlation between TA-G concentration in the latex and main roots was analyzed with a linear model.

To investigate the TA-G concentration of main roots, latex-drained main roots and laticifer-free main root cortex, we grew 16 plants from genotype *A34* for 12 wk. To harvest drained and nondrained roots, the main roots of 8 plants were cut 2 cm below the tiller into two 1 mm slices. From one slice, the latex was collected using filter paper (Whatman 40) before freezing it in liquid nitrogen (“drained”). The other slice was flash-frozen without collecting latex (“nondrained”). To harvest the laticifer-free cortex tissue, the outer cortex zones of the main roots of 8 plants were dissected with a razor blade and frozen in liquid nitrogen. All samples were ground to a fine powder, weighed, extracted, and analyzed for TA-G concentrations as described above. Differences in the TA-G concentration between root samples were analyzed with a one-way ANOVA. Pairwise comparisons were performed with a Tukey posthoc test.

### Transcriptome Sequencing

To identify putative germacrene A synthases, we sequenced the transcriptome of *T*. *officinale* main root and latex using Illumina HiSeq 2500. The main roots of six 10 wk-old plants from genotype *A34* were cut, the exuding latex was collected into 100 μl homogenization buffer [69] (4 M guanidine isothiocyanate, 100 mM Tris-HCl, pH 7.0, and 5 mM dithiothreitol) and the latex as well as main root samples were frozen in liquid nitrogen. Main root samples were ground to a fine powder and RNA was extracted from 100 mg tissue with the RNAeasy plant mini kit (Qiagen) following the manufacturer’s instructions. For latex RNA extraction, 900 μl QIAzol lysis reagent was added to the latex samples, vortexed, and RNA isolated using RNAeasy Plant Lipid Tissue Mini Kit (Qiagen) following the standard procedure. On-column DNA digestion for main root and latex samples was performed using DNase free RNase (Qiagen). The six samples for main root and latex were pooled equimolarly. TruSeq RNA-compatible libraries were prepared and PolyA enrichment was performed before sequencing the two transcriptomes on an Illumina HiSeq 2500 with 20 Mio reads per library, 100 base pair, paired end. De novo transcriptome assembly on pooled reads from main root and latex sample was performed using Trinity (version Trinityrnaseq_r20131110) [70,71] running at default settings. Raw reads were deposited in the NCBI Sequence Read Archive (SRA) (BioProject Accession PRJNA301484).

### Identification and Phylogenetic Analysis of *T*. *officinale* Germacrene A Synthases

To identify putative germacrene A synthase genes in the *T*. *officinale* transcriptome, we performed a BLAST analysis using the amino acid sequences of two known germacrene A synthases from chicory as templates [46]. Two putative germacrene A synthase genes were identified and designated as *ToGAS1* and *ToGAS2*. Sequences were deposited in GenBank with the accession numbers KT898039 (*ToGAS1*) and KT898040 (*ToGAS2*). For the estimation of a phylogenetic tree of ToGAS1, ToGAS2, and characterized terpene synthases from other Asteraceae (S4 Table), we used the MUSCLE algorithm (gap open, −2.9; gap extend, 0; hydrophobicity multiplier, 1.5; clustering method, upgmb) implemented in MEGA5 [72] to compute an amino acid alignment using a neighbor-joining algorithm (Poisson model). All positions with less than 80% site coverage were eliminated. A bootstrap resampling analysis with 1,000 replicates was performed to evaluate tree topology.

### Cloning and Heterologous Expression of Germacrene A Synthases ToGAS1/2

The two putative germacrene A synthases were heterologously expressed in *E*. *coli* to verify their biochemical function. The complete open reading frames (S2 Text) encoding putative proteins with 559 amino acids for ToGAS1 and 583 amino acids for ToGAS2 could be amplified from root cDNA using the primers GAS1fwd (ATGGCAGCAGTTGAAGCCAATGGG) and GAS1rev (TTACATGGGCGAAGAACCTACA) for ToGAS1 and the primers GAS2fwd (ATGGCTCTAGTTAGAAACAACAGTAG) and GAS2rev (TCAGTTTTCGAGACTCGGTGGAGGAC) for ToGAS2. The genes were cloned into the vector pET100/D-TOPO (Invitrogen, Carlsbad, CA, USA) and an *E*. *coli* strain BL21 Codon Plus (Invitrogen) was used for heterologous expression. Expression was induced by addition of isopropyl-1-thio-D-galactopyranoside to a final concentration of 1 mM. The cells were collected by centrifugation at 4,000 g for 6 min and disrupted by a 4 × 30 sec treatment with a sonicator in chilled extraction buffer (50 mM Mopso, pH 7.0, with 5 mM MgCl_2_, 5 mM sodium ascorbate, 0.5 mM phenylmethanesulfonylfluoride, 5 mM dithiothreitol and 10% v/v glycerol). The cell fragments were removed by centrifugation at 14,000 g, and the supernatant was desalted into assay buffer (10 mM Mopso, pH 7.0, 1 mM dithiothreitol, 10% v/v glycerol) by passage through an Econopac 10DG column (BioRad, Hercules, CA, USA). Enzyme assays were performed in a Teflon-sealed, screw-capped 1 ml GC glass vial containing 50 μl of the bacterial extract and 50 μl assay buffer with 10 μM (*E*,*- E*)-FPP, 10 mM MgCl_2_, 0.2 mM NaWO_4_ and 0.1 mM NaF. An SPME (solid phase microextraction) fiber consisting of 100 μm polydimethylsiloxane (SUPELCO, Belafonte, PA, USA) was placed into the headspace of the vial for 60 min incubation at 30°C and then inserted into the injector of the gas chromatograph for analysis of the adsorbed reaction products. GC-MS analysis was conducted using an Agilent 6890 Series gas chromatograph coupled to an Agilent 5973 quadrupole mass selective detector (interface temp, 250°C; quadrupole temp, 150°C; source temp, 230°C; electron energy, 70 eV). The GC was operated with a DB-5MS column (Agilent, Santa Clara, USA, 30 m x 0.25 mm x 0.25 μm). The sample (SPME) was injected without split at an initial oven temperature of 50°C. The temperature was held for 2 min, then increased to 240°C with a gradient of 7°C*min^−1^, and further increased to 300°C with a gradient of 60°C*min^−1^ and a hold of 2 min. For the GC-MS analysis with a cooler injector, the injector temperature was reduced from 220°C to 150°C. Chiral GC-MS analysis was performed using a R-βDEXsm-column (Restek, Bad Homburg, Germany) and a temperature program from 50°C (2 min hold) at 2°C*min^−1^ to 220°C (1 min hold). A (+)-germacrene A synthase (MrTPS3) from chamomile (*Matricaria recutita*) [73] was used to prepare an authentic (+)-germacrene A standard.

### Expression Analysis of *ToGAS1* and *ToGAS2*

To measure the expression of *ToGAS1* and *ToGAS2*, we harvested latex, main roots and outer cortex cells of 8 wk-old *A34* plants. Plants we cultivated in a growth chamber at 18°C and 75% humidity with a 16-h photoperiod (250 μmol m^−2^ s^−1^) in 50% Ricoterlanderde (RICOTER Erdaufbereitung AG, Aarberg, Switzerland), 40% sphagnum peat and 10% sand. Plants were fertilized every week with 0.1% Plantaktiv 16 + 6 + 26 Typ K (Hauert HBG Dünger, Grossaffoltern, Switzerland) according to the manufacturer`s instructions. Total RNA was isolated from roots using the GeneJET Plant RNA Purification Mini Kit (Thermo Scientific) according to the manufacturer’s instructions. Total RNA was isolated from latex by dissecting the main root with a razor blade and harvesting 10 μl of expelling latex in 100 μl homogenization buffer (see above). After the addition of 900 μl QIAzol Lysis Reagent, RNA was isolated using the RNeasy Lipid Tissue Mini Kit (Qiagen) according to the manufacturer’s instructions. All RNA samples were treated on column with RNase-free DNase I (Qiagen), and the RNA quality and quantity was determined on agarose gels as well as by spectrophotometric analysis using a ND-1000 spectrophotometer (NanoDrop Technologies).

From each sample, 1 μg total RNA was used for reverse transcription using oligo(dT) primers and SuperScript II Reverse Transcriptase (Invitrogen) according to the manufacturer’s instructions. The cDNA quality was determined by PCR using the primer combination ToActin-fwd (5`-CGTGACATCAAGGAGAAGC-3`) und ToActin-rev (5`-GCTTGGAGATCCACATCTG-3`). Quantitative real-time PCR (qRT-PCR) was performed according to the Minimum Information for Publication of Quantitative Real-Time PCR Experiments (MIQE) guidelines [74]. All primer sequences were validated in silico (Oligo Proberty Scan, Eurofins MWG, http://www.mwg-biotech.com) and accepted when they yielded single amplicons as it was proven by melt curve analysis, agarose gel electrophoresis, and sequencing.

qRT-PCR primers for ToGAS1 (ToGAS1-fwd, 5`-AAATTTCCCTCCTTCAGTATGGGG-3`; ToGAS1-rev, 5`-CTTATTGGAATCCATGGTTGGATCTAC-3`) and ToGAS2 (ToGAS2-fwd, 5`-CTGATACTACCATTGATGCAACCAC-3`; ToGAS2-rev, 5`-CAGCATCAATCTCTTCTGGATAAAG-3`) were designed to anneal at positions of significant sequence divergence between these two *GAS* genes to yield specific products. The *T*. *officinale* transcription elongation factor encoding *EF-1α* gene was used as a reference and amplified with the primer combination ToEF1α-fwd (5`-ACTGGTACTTCCCAGGCCGATTGC-3`) and ToEF1α-rev (5`-TTGTTTCACACCAAGGGTGAAGGCG-3`). qRT-PCR experiments were carried out with the LightCycler 96 Real-Time PCR System (Roche Diagnostics International Ltd) using the KAPA SYBR FAST qPCR Kit (Kapa Biosystems) according to the manufacturer’s instructions. For each experiment, three biological replicates were performed with two technical replicates for each biological triplicate. Relative gene expression levels were calculated with the LightCycler 96 Application Software (Version 1.1.0.1320, Roche Diagnostics International Ltd). Expression between different tissues and genes was analyzed with a two-way ANOVA, and pairwise comparison of the expression levels of the two genes performed with Tukey posthoc test.

### RNAi

Based on the transcriptome data, we targeted *ToGAS1* by RNAi. For silencing, we used the triploid genotype *A34* from the above-mentioned 17 *T*. *officinale* genotype based on transformation compatibility and intermediate levels of TA-G concentration. *A34* is a triploid, synthetic apomict created by crossing a diploid mother from France with diploid pollen from a triploid apomict from the Netherlands [75]. For the construction of the germacrene A synthase RNAi vector, a 191-bp germacrene A synthase PCR fragment was amplified from *T*. *officinale* leaf cDNA using the RNAi-dicer optimized primers ToGermA-RNAi-BamHI_fw (5’-aaaGGATCCGGGATAGAGTACCAGAGATT-3’) and ToGermA-RNAi-XhoI_rev (5’-aaaCTCGAGGGCACTAATGTCCCACCTA-3’). This fragment was digested with BamHI and XhoI and inserted into the respective sites of the Gateway vector pENTR4 (Invitrogen). The resulting vector was used for LR recombination (mediated by LR clonase, Invitrogen) with the GW-compatible destination vector pFGC5941 (http://www.chromDB.org), which contains the CaMV 35S promoter and the chalcone synthase intron from *Petunia hybrida*. The integrity of the constructs was verified by sequencing and subsequently used for *Agrobacterium tumefaciens*-mediated stable transformation of the *T*. *officinale A34* genotype using the same method as described previously [76].

### Screening and Characterization of Transgenic Lines

The T1 generation of 13 transformed lines was screened for latex secondary metabolite concentrations using three individuals of each line. Main root latex of 8 wk-old *T*. *officinale* was collected into Eppendorf tubes and frozen in liquid nitrogen. Latex was extracted as described above using 1 ml methanol containing 10 μg*ml^−1^ loganin as internal standard. Samples were analyzed on HPLC-DAD as described above. Five lines were selected for further molecular and phenotypic characterization. First, the transgenic lines were confirmed to be triploid by flow cytometry. Second, the insertion of the transgene was verified by PCR and sanger sequencing on genomic DNA using the primer combination P2 + ToGermA-RNAi-XhoI_rev and P3 + ToGermA-RNAi-XhoI_rev, with 5‘-TACCTTCCCACAATTCGTCG-3‘f for P2, 5‘-CAGGTATTGGATCCTAGGTG-3‘ for P3 and 5‘-AAACTCGAGGGCACTAATGTCCCACCTA-3’ for ToGermA-RNAi-XhoI_rev. Third, transcript levels of *ToGAS1* and *ToGAS2* were determined in the T2 generation by qPCR using the primers described above. Four individuals of the TA-G-deficient (RNAi-1, -12b, -16) and control (RNAi-9, -15, WT) lines grown in soil were harvested at an age of 8 wk. Main root tissue was frozen in liquid nitrogen and ground under liquid nitrogen to a fine powder. RNA was extracted using the GeneJET Plant RNA Purification Mini Kit (Thermo Scientific) according to the manufacturer’s instructions. RT-qPCR was performed for *ToGAS1*, *ToGAS2* and *ToEF1α* as described above (*n* = 4). Relative gene expression levels were calculated with the LightCycler 96 Application Software. Gene expression was analyzed with generalized linear models using a gamma error distribution for *ToGAS1* and a Gaussian error distribution for *ToGAS2*. Forth, we determined latex fresh mass, latex secondary metabolites, total protein, amino acid and sugar concentrations in the roots. To analyze concentration of TA-G and total PIEs in the transgenic plants, we harvested six individuals of three TA-G-deficient (RNAi-1, -12b, -16) and three control (wild type, RNAi-9, -15) lines in the T2 generation at an age of 8 wk. Main root latex was collected into Eppendorf tubes, which were flash-frozen in liquid nitrogen. 1 ml methanol containing 10 μg*ml^−1^ loganin and 100 μg*ml^−1^ salicin as internal standards for TA-G and PIEs, respectively, were added to the Eppendorf tubes. Samples were extracted and analyzed as described above. Differences in the latex fresh mass, as well as in the concentration of TA-G and total PIEs between TA-G-deficient and control lines, were analyzed with one-way ANOVAs. To determine whether total TritAc concentration was affected by silencing, we collected main root latex from 6 individuals of 9 wk-old TA-G-deficient (RNAi-1, -12b, -16) and control lines (wild type, RNAi-9, 15) into glass vials, which were immediately frozen in liquid nitrogen. Samples were extracted with 1 ml hexane containing 100 μg*ml^−1^ cholesteryl acetate as internal standard. Samples were processed and analyzed on GC-FID as described above. Differences in the concentration of total TritAcs were analyzed with a one-way ANOVA.

To determine soluble protein, free amino acid and soluble sugar concentrations in the roots of the TA-G-deficient (RNAi-1, -12b, -16) and control (wild type, RNAi-9, -15) lines, we harvested 5 individuals of each line at an age of 12 wk. Root systems were exposed, washed, and main and side roots frozen in liquid nitrogen. Root tissue was ground under liquid nitrogen to a fine powder. For extraction, 1 ml 0.1 M TRIS-HCl, pH = 7.0 was added to 100 mg ground tissue, vortexed and centrifuged at room temperature at 17,000 g for 10 min. The supernatant was stored at −20°C until analysis.

Soluble protein concentration was determined using the Bradford assay and quantified using a standard curve of albumin [77]. Differences in soluble protein concentrations between TA-G-deficient and control lines, as well as between root tissues, were analyzed with two-way ANOVAs. To determine free amino acid concentrations, 10 μl of the diluted samples were mixed with 90 μl ^13^C, ^15^N labelled amino acid mix (20 μg amino acids*ml^−1^) (Isotec, Miamisburg, USA) and 100 μl borate buffer (0.9 M, pH = 10.0). To derivatize amino acids, 22 μl 30 mM fluorenylmethoxy-carbonyl chloride was added and samples were vortexed. After 5 min, 800 μl hexane was added to stop the reaction; the samples were vortexed and placed at room temperature until phases had separated. The lower, aqueous phase was analyzed on an Agilent 1200 HPLC system coupled to an API 5000 tandem mass spectrometer according to [78]. To determine soluble sugar concentrations, main root samples were diluted 1:10 and side root samples 1:5 in 0.1 M TRIS-HCl, pH = 7.0. Samples were analyzed on an Agilent 1200 HPLC system (Agilent Technologies, Germany) coupled to an API 3200 tandem mass spectrometer (Applied Biosystems, Germany) equipped with a turbospray ion source operating in negative ionization mode. Injection volume was 1 μl. Metabolite separation was accomplished by an apHera NH_2_, 15 cm x 4.6 mm x 3 μm. The mobile phase consisted of water (A) and acetonitrile (B) utilizing a flow of 1 ml*min^−1^ with the following gradient: 0 min: 20% A, 0.5 min: 20% A, 13 min: 45% B, followed by column reconditioning. The column temperature was maintained at 20°C. The ion spray voltage was maintained at −4.5 keV. The turbo gas temperature was set at 600°C. Nebulizing gas was set at 50 psi, curtain gas at 20 psi, heating gas at 60 psi and collision gas at 5 psi. Multiple reaction monitoring (MRM) was used to monitor analyte parent ion → product ion: m/z 178.9 →89 (collision energy (CE) −10 V; declustering potential (DP) −25 V), for glucose; m/z 178.9 →89 (CE −12V; DP −25V) for fructose; m/z 341.03 →58.96 (CE -52V; DP -45V) for sucrose; Both Q1 and Q3 quadrupoles were maintained at unit resolution. Analyst 1.5 software (Applied Biosystems, Darmstadt, Germany) was used for data acquisition and processing. All compounds were identified based on comparison of retention times and mass spectra to those of commercial standards. Glucose, fructose, and sucrose concentrations were quantified using external standard curves obtained from commercial standards. Differences in the sugar concentrations between TA-G-deficient and control lines, as well as between root tissues, were analyzed with two-way ANOVAs.

### No-Choice Experiment with *M*. *melolontha* and Transgenic *T*. *officinale*

To investigate whether silencing of *ToGAS1* affects plant performance, we measured root and leaf mass of three TA-G-deficient (RNAi-1, -12b, -16) and three control (wild type, RNAi-9, -15) lines. For each line, 24 plants of the T2 generation were cultivated for 8 wk. Half of the plants were infested with one preweighed *M*. *melolontha* larva. One week after infestation, plants were separated into side roots, main roots and leaves, and plant material was dried for three days at 60°C before weighing. As TA-G-deficient lines had 50% lower root mass than control lines, resistance was expressed relative to the control plants of each genotype (100*(1 − (mass herbivore plant / mean mass control plants of its genotypes))) and analyzed with Kruskal-Wallis rank sum tests.

### Choice Experiment with *M*. *melolontha* and Transgenic *T*. *officinale*

In order to test *M*. *melolontha* preference for and plant resistance of TAG-deficient and wild type *T*. *officinale* plants, three TA-G-deficient (RNAi-1, -12b, -16) and three control (wild type, RNAi-9, -15) lines were tested in a choice experiment with *M*. *melolontha* larvae. Larvae were starved for three days prior to the experiment. Each larva was placed into a 180 ml plastic beaker, which was filled with 2–3 mm vermiculite. The roots of 5 wk-old *T*. *officinale* seedlings of the T2 generation (grown in soil in seedling trays) were washed, briefly dried with a tissue and the mass of the plants determined. One TA-G-deficient and one control plant was embedded into the vermiculite-filled beaker at opposite edges, with 9 replicates of each possible pairwise combination. Larval feeding site was scored visually after 3 h by inspecting the beakers from the outside. To determine root mass consumption, plants were recovered after 4 h. The plants were separated into shoots and roots and dried for three days at 60°C. Fresh mass was calculated from dry mass using a common conversion coefficient based on the fresh/dry mass ratio of five non-manipulated seedlings of each genotype. Root mass consumption was analyzed using paired Student’s *t* tests. To obtain sufficiently large sample sizes for a binomial test, larval preference was analyzed by pooling the data for the three TA-G-deficient and control lines. In order to test whether differences in primary metabolites affected *M*. *melolontha* choice, we correlated *M*. *melolontha* preference and root mass consumption to total main root protein concentration as determined from 12 wk-old plants as described above. Data were analyzed with Pearson’s product–moment correlation.

### Choice Experiment with Carrot Seedlings Painted with TA-G-Deficient and Wild Type Latex

To test whether *M*. *melolontha* preference for TA-G-deficient *T*. *officinale* is mediated by latex metabolites, we recorded larval choice among carrot seedlings painted with latex of three TA-G-deficient (RNAi-1, -12b, -16) and three control (RNAi-9, -14, -15) lines. *M*. *melolontha* larvae were starved for two days. Each larva was placed into the center of a 180 ml plastic beaker, which was filled with 2–3 mm vermiculite. The roots of the 6 wk-old carrot seedlings were completely covered with latex of 5 mo-old TA-G-deficient and control *T*. *officinale* of the T1 generation, cultivated in 21 pots in soil (identical growth conditions as described above, except light source from NH 360 FLX SUNLUX ACE Japan). Seedlings painted with TA-G-deficient and control latex were pairwise arranged on opposite edges of the beaker, resulting in 6–11 replicates of each possible pairwise combination. Larval feeding site was visually scored after three hours. Larval preference was analyzed based on pooled data for the three TA-G-deficient and control lines using a binomial test.

### Choice Experiments with Purified TA-G

To determine physiologically relevant TA-G concentrations for bioassays, we analyzed TA-G concentration from latex, main, and side roots and leaves from three wild type *A34* plants. Main root latex of 11 wk-old *T*. *officinale* was collected into Eppendorf tubes, frozen in liquid nitrogen and extracted with 1 ml methanol containing 10 μg*ml^−1^ loganin as an internal standard as described above. Main roots, side roots, and leaf tissues were flash-frozen in liquid nitrogen and ground to fine powder. 100 mg tissue was extracted with 1 ml methanol containing 10 μg*ml^−1^ loganin, vortexed, centrifuged, and the supernatant transferred to HPLC vials. All samples were analyzed as described above for the analysis of TA-G in the main roots. Peak area was integrated at 245 nm for TA-G and normalized to loganin as internal standard.

To test whether TA-G deters *M*. *melolontha*, we isolated TA-G from latex and added it to artificial diet at a concentration of 3 μg TA-G*mg^−1^ diet. TA-G was isolated using 300 ml latex methanol extracts obtained from 300 *A34* plants grown in the greenhouse. 10 ml water was added to the methanol extract before methanol was completely evaporated using rotary-evaporation. The aqueous solution was loaded on a Sephadex LH20 (GE-Healthcare, Germany) column with 2.5 cm x 30 cm dimensions. The compounds were eluted from the column using water at a flow speed of 1 ml*min^−1^. 15 ml fractions were collected and analyzed for TA-G on an Agilent 1200 HPLC system (Agilent Technologies, Germany) coupled to an API 3200 tandem mass spectrometer (Applied Biosystems, Germany) equipped with a turbospray ion source operating in negative ionization mode. Injection volume was 5 μl using flow injection analysis. The mobile phase consisted of 0.05% formic acid (A) and acetonitrile (B) utilizing a flow of 1 ml*min^−1^. 50% A was maintained for 0.5 min. The column temperature was kept at 20°C. The ion spray voltage was maintained at −4.5 keV. The turbo gas temperature was set at 600°C. Nebulizing gas was set at 50 psi, curtain gas at 30 psi, heating gas at 60 psi and collision gas at 5 psi. Multiple reaction monitoring (MRM) was used to monitor analyte parent ion → product ion: m/z 423 →261 (collision energy (CE) −14 V; declustering potential (DP) -40 V), for TA-G; m/z 447 →151 (CE -26V; DP -100V) for di-PIEs; m/z 581 →151 (CE -38V; DP -140V) for tri-PIEs. Both Q1 and Q3 quadrupoles were maintained at unit resolution. Analyst 1.5 software (Applied Biosystems, Darmstadt, Germany) was used for data acquisition and processing.

Pure fractions were pooled and lyophilized using an Alpha 1–4 LD plus freeze dryer (Martin Christ GmbH, Germany). 30 *M*. *melolontha* larvae were starved for two days before providing them 300 mg artificial diet supplemented with either 30 μl 30 mg*ml^−1^ TA-G or with 30 μl water as solvent control (artificial diet: 25 g bean flower, 2.4 g Agar from Roth, Agar-Agar bacteriologist, 105 ml tap water, 33.3 g cooked and mashed carrots). Larvae were allowed to feed for 24 h inside a 180 ml plastic beaker covered with a moist tissue before the remaining food was weighed. Food consumption was analyzed using Student’s *t* test. Larvae that consumed less than 30 mg diet were considered inactive and were excluded from the analysis.

### Common Garden Experiment

In order to examine the effects of latex secondary metabolites on plant resistance in the field, we cultivated 2,400 *T*. *officinale* from the above-mentioned 20 genotypes in a common garden with and without *M*. *melolontha* infestation over one year at a field site in Jena, Germany (50°54'34.8"N; 11°34'00.1"E). Seeds were surface sterilized, germinated on moist filter paper in petri dishes in spring 2013, and the emerging seedlings were transferred onto peat balls after 10 d. One month after germination, seedlings were conditioned outside for one week before planting them into the field site. At the field site, the top 50 cm soil layer was removed and a metal mesh installed on the ground to confine vertical *M*. *melolontha* movement. Experimental units (“plots”) were set up using 20 circular plastic tubes (50 cm depth, 2 m diameter) that were placed on the top of the mesh and filled up with the original soil. One wheelbarrow of peat was mixed with the top 20 cm of soil to facilitate plant growth. In each plot, 6 replicates of all 20 genotypes were placed randomly in a quadratic grid with 10 cm distance between plants. To buffer edge effects, these experimental plants were surrounded with an additional row of *T*. *officinale* plants, which were excluded from data analysis. Plants were watered as necessary during the first two months after planting and plots weeded monthly. Three weeks after planting, the length of the longest leaf (“maximal leaf length”) was measured for each plant. Subsequently, 72 late L2 or early L3 *M*. *melolontha* larvae were homogenously distributed in half of the plots (“herbivory”), while the remaining plots were not manipulated (“control”). As a nondestructive measurement of plant performance, we measured maximal leaf length—a reliable predictor for above and below ground biomass under greenhouse conditions (S19 Fig)—of each plant every month until the end of the growing season.

For statistical analysis, the length of the longest leaf at the beginning of the experiment was subtracted from the maximal leaf length measured each month to reduce the impact of initial differences in plant size (“leaf growth”). To normalize between genotypes, leaf growth of herbivore-treated plants was expressed relative to control plants of the same genotype (“relative leaf growth”).

$$Relative\;leaf\;growth\;(j)=\frac{Mean(Max\;leaf\;length\;H(ij)-Initial\;max\;leaf\;length\;H(ij))}{Mean(Max\;leaf\;length\;C(ij)-Initial\;max\;leaf\;length\;C(ij))}$$

with H = herbivore-infested plants

C = control plant

i = individual plant

j = genotype

Initial max leaf length = maximal leaf length in June before infestation

Correlations between relative leaf growth and TA-G, total PIEs, total TritAcs, latex fresh mass and total TA-G (latex mass * TA-G concentration) were performed based on mean values for each genotype for each month separately with Pearson’s product–moment correlations in R. The combined effect of latex mass and TA-G concentration on relative leaf growth was analyzed with a multiple linear regression. Secondary metabolite concentrations and latex fresh mass were obtained from the experiment with the 20 genotypes in the greenhouse as described above. Three genotypes lacking TA-G were excluded from the analysis due to the presence of unknown and thus unquantifiable sesquiterpene lactone glycosides.

In order to test whether damage caused by *M*. *melolontha* in the field is proportional to plant size, we assessed the correlation between leaf length of herbivore-infested individuals and leaf length of non-infested individuals of the 17 genotypes with Pearson’s product-moment correlations.

To correlate secondary metabolite concentrations to reproductive plant fitness, the number of flowers was counted every month in the following year. Correlations between relative number of flowers (number of flowers of the herbivore-infested plants expressed relative to noninfested plants of each genotype) and TA-G, total PIEs, and total TritAcs were analyzed with linear models and Pearson product–moment correlations based on the mean value of each of the 17 genotypes. Difference in TA-G concentration between genotypes that flowered and genotypes that did not flower at the beginning of the flowering season was analyzed with a Wilcoxon rank sum test based on the mean value of each of the 17 genotypes.

## Supporting Information

S1 Data Data used to create figures and summary tables. (XLSX)

S1 Fig Correlation between TA-G concentration and latex fresh mass across 17 *T*. *officinale* genotypes. Latex fresh mass was determined by cutting the main roots 1 cm below the tiller and collecting the exuding latex. One data point represents the mean of one genotype. The *p*-value of a linear model is shown. Underlying data can be found in S1 Data.
(TIF)

S2 Fig TA-G is not induced after 11 d of *M*. *melolontha* herbivory. TA-G concentration was measured from 17 *T*. *officinale* genotypes with and without *M*. *melolontha* herbivory. The *p*-value of a *t* test is shown. Underlying data can be found in S1 Data.
(TIF)

S3 Fig Correlation between TA-G concentrations of *M*. *melolontha*-infested and noninfested *T*. *officinale* plants. One data point represents the mean of one genotype. *p*-Value and r^2^ value of a linear model are shown. Underlying data can be found in S1 Data.
(TIF)

S4 Fig TA-G predominantly accumulates in the latex. Statistics of one-way ANOVAs are shown. Different lower case letters indicate significant differences in TA-G concentrations according to Tukey posthoc tests. Underlying data can be found in S1 Data.
(TIF)

S5 Fig Correlation between TA-G concentrations in latex and main roots across 17 *T*. *officinale* genotypes. One data point represents the mean of one genotype. *p*-Value and r^2^ values of a linear model are shown. Underlying data can be found in S1 Data.
(TIF)

S6 Fig Heterologous expression of germacrene A synthases (ToGAS1/2) in *E*. *coli*. a. GC-MS analysis of enzyme products from recombinant ToGAS1 and ToGAS2 incubated with the substrate FDP. Germacrene A produced by ToGAS1 and ToGAS2 is converted to β-elemene during hot GC injection. cont, contamination. b. Chiral analysis of recombinant ToGAS1 and ToGAS2 enzyme products. Retention times and mass spectra of ToGAS enzyme products were compared to those of (-)-β-elemene obtained as a thermal rearrangement product of (+)-germacrene A synthesized by MrTPS3 from chamomile [73]. c. GC-MS analysis of monoterpene products from recombinant ToGAS1 and ToGAS2 incubated with the substrate geranyl diphosphate (GPP). 1, myrcene; 2, limonene; 3, (*Z*)-β-ocimene; 4, (*E*)-β-ocimene; 5, terpinolene; 6, linalool; 7, α-terpineol. IC = ion count.
(TIF)

S7 Fig Latex fresh mass of transgenic TA-G deficient lines and control *T*. *officinale* lines. Latex fresh mass was determined by cutting the main roots 1 cm below the tiller and collecting the exuding latex. Statistics of a one-way ANOVA is shown. Underlying data can be found in S1 Data.
(TIF)

S8 Fig Silencing efficiency of *ToGAS1/2*. Expression of *ToGAS1* and *ToGAS2* of the TA-G-deficient (RNAi-1, -12b, 16) and control (RNAi-9, -15, WT) lines normalized to the elongation factor *ToEF1α*. Different letters indicate significant differences in expression of *ToGAS1* (lower case) and *ToGAS2* (upper case) between the different lines in a generalized linear model. *n* = 4. Underlying data can be found in S1 Data.
(TIF)

S9 Fig Vegetative biomass of TA-G-deficient and control *T*. *officinale* lines at an age of 8 wk. *X*-axis shows individual silenced lines. Statistics show one-way ANOVA. Sum Sq = sum of squares. N = 12. Underlying data can be found in S1 Data.
(TIF)

S10 Fig Relative leaf mass of TA-G-deficient and control *T*. *officinale* lines upon *M*. *melolontha* attack. Larvae fed for 7 d on 8 wk-old *T*. *officinale* seedlings. Relative leaf mass is the mass of each herbivore infested plant relative to the mean leaf mass of the control plants of its genotype. Statistics from Kruskal-Wallis rank sum test is shown. *n* = 12. Underlying data can be found in S1 Data.
(TIF)

S11 Fig Vegetative biomass of TA-G-deficient and control *T*. *officinale* lines at an age of 5 wk. *X*-axis shows individual silenced lines. Statistics show one-way ANOVA. Sum Sq = sum of squares. *n* = 5. Underlying data can be found in S1 Data.
(TIF)

S12 Fig Concentration of soluble proteins in roots of TA-G-deficient and control *T*. *officinale*. Eight week-old *T*. *officinale* were analyzed. *X*-axis shows individual silenced lines. Statistics of two-way ANOVA is shown. Sum Sq = sum of squares. *n* = 6. Underlying data can be found in S1 Data.
(TIF)

S13 Fig Concentrations of free amino acids in roots of TA-G-deficient and control *T*. *officinale*. Eight week-old *T*. *officinale* were analyzed. Data from the three TA-G-deficient (RNAi-1, -12b, -16) and control lines (wild type, RNAi-9, RNAi-15) were pooled. *n* = 6. Underlying data can be found in S1 Data.
(TIF)

S14 Fig Concentrations of soluble sugars in roots of TA-G-deficient and control *T*. *officinale*. Eight week-old *T*. *officinale* were analyzed. *X*-axis shows individual silenced lines. Statistics of two-way ANOVA is shown. Sum Sq = sum of squares. *n* = 6. Underlying data can be found in S1 Data.
(TIF)

S15 Fig Correlations of soluble protein concentration and *M*. *melolontha* choice across TA-G deficient and control lines. *p*-Values from Pearson product–moment correlations are shown. Underlying data can be found in S1 Data.
(TIF)

S16 Fig TA-G and total PIE concentrations in latex of TA-G-deficient and control *T*. *officinale* lines. Latex of 8 wk-old *T*. *officinale* was analyzed. *X*-axis shows individual silenced lines. TA-G = taraxinic acid β-D-glucopyranosyl ester; PIE = phenolic inositol ester. Statistics of one-way ANOVA is shown. *n* = 6. Underlying data can be found in S1 Data.
(TIF)

S17 Fig Total triterpene acetate concentrations in latex of TA-G-deficient and control *T*. *officinale*. Latex of 8 wk-old *T*. *officinale* was analyzed. *X*-axis shows individual silenced lines. Statistics of one-way ANOVA is shown. *n* = 6. Underlying data can be found in S1 Data.
(TIF)

S18 Fig Overview of common garden experiment. Note that container logo and number in the lower right corner have been removed during post processing of this photograph.
(TIF)

S19 Fig Correlation between leaf length and vegetative biomass. Correlation between leaf length and leaf and root dry mass across three genotypes (*19*.*31*, *2*.*8A*, *A34*) over a growth period of 5 wk. Five plants per genotype were harvested every week starting with 6 wk-old plants cultivated in a growth chamber. Each data point represents the mean of each genotype and time point. Statistics from linear models are shown. Underlying data can be found in S1 Data.
(TIF)

S20 Fig Correlation between TA-G and leaf growth in a common garden experiment with and without *M*. *melolontha* infestation. TA-G concentration tended to be positively correlated to leaf growth (maximal leaf length of each month–maximal leaf length before infestation) under *M*. *melolontha* attack and negatively correlated to leaf growth in the control treatment towards the end of the growing season. Plants were infested in June. Each data point represents the mean of one genotype. *p*-Values from Pearson’s product–moment correlations based on mean values of each genotype are shown. Underlying data can be found in S1 Data.
(TIF)

S21 Fig Correlations of relative leaf growth with total concentrations of PIEs and TritAcs in a common garden. Relative leaf growth is the size increase of the longest leaf of the herbivore-infested plants compared of the size of the longest leaf before infestation, expressed relative to the leaf growth of the control plants of each genotype. Each data point represents the mean relative leaf growth of one *T*. *officinale* genotype. Plants were infested in June. *p*-Values from Pearson’s product–moment correlations based on mean values of each genotype are shown. Underlying data can be found in S1 Data.
(TIF)

S22 Fig Correlation between average leaf length of *M*. *melolontha*-infested plants and mean leaf length of non-infested plants in the common garden experiment in September. Herbivore damage was proportional to plant size. The *p*-value of a Pearson product–moment correlation is shown. One data point represents the mean of one genotype. Underlying data can be found in S1 Data.
(TIF)

S23 Fig Picture of *M*. *melolontha* feeding on *T*. *officinale* roots. (TIF)

S24 Fig In-source fragmentation pattern of TA-G and putative sesquiterpene lactone glycosides. A. In-source fragmentation pattern of TA-G, obtained from a latex methanol extract of genotype *A34*. B–D. Putative sesquiterpene lactone glycosides. A latex methanol extract from genotype 17.20A was screened for fragmentation patterns resembling TA-G. All samples were analyzed on an Esquire 6000 ESI-Ion Trap mass spectrometer in positive ionization mode [36].
(TIF)

S1 Table Origin of 20 *T*. *officinale* genotypes. Genotype *A34* is a triploid, synthetic apomict, created by crossing a sexual diploid mother from France with diploid pollen from a triploid apomict from the Netherlands [75].
(DOCX)

S2 Table Multiple linear regression of *M*. *melolontha* growth, TA-G concentration and latex fresh mass after 11 d of larval feeding on 17 *T*. *officinale* genotypes. (DOCX)

S3 Table Linear regression of *M*. *melolontha* mass gain and total amount of TA-G (TA-G concentration * latex mass) after 11 d of larval feeding on 17 *T*. *officinale* genotypes. (DOCX)

S4 Table Accession numbers of protein sequences used for dendrogram analysis of Asteraceae terpene synthases. (DOCX)

S5 Table *p*-Values of Pearson’s product–moment correlations between relative leaf growth and latex mass as well as between relative leaf growth and total TA-G (TA-G concentration * latex mass) across 17 *T*. *officinale* genotypes in the common garden experiment. Leaf growth is the increase in maximal leaf length compared to maximal leaf length before infestation.
(DOCX)

S6 Table Multiple linear regressions of relative leaf length, TA-G concentration and latex fresh mass across 17 *T*. *officinale* genotypes in the common garden field experiment. Relative leaf growth is the mean leaf growth of herbivore-infested plants of each genotype during the infestation period compared to the mean leaf growth of the control plants of each genotype (leaf growth: increase in maximal leaf length compared to maximal leaf length before infestation). Std. Error = Standard error.
(DOCX)

S7 Table Density of *M*. *melolontha* per m^2^ in common garden field experiment at the end of the flowering season in the second year. Initial density of *M*. *melolontha* in the herbivory treatment was 23 *M*. *melolontha* larvae per m^2^.
(DOCX)

S1 Text Selection procedure of 20 *T*. *officinale* genotypes. (DOCX)

S2 Text Full length sequences of ToGAS1 and ToGAS2. (DOCX)

## Abbreviations

FDP: farnesyl diphosphate
GC-FID: gas chromatography-flame ionization detector
LC-MS: liquid chromatography-mass spectrometry
PIE: phenolic inositol ester
RNAi: RNA interference
TA-G: taraxinic acid β-D-glucopyranosyl ester
TritAcs: triterpene acetates
