# Supplementary figures and images for: A Latex Metabolite Benefits Plant Fitness under Root Herbivore Attack
Source: PLoS Biol. 2016 Jan 5;14(1):e1002332. doi: 10.1371/journal.pbio.1002332 (PMC4701418; doi:10.1371/journal.pbio.1002332)

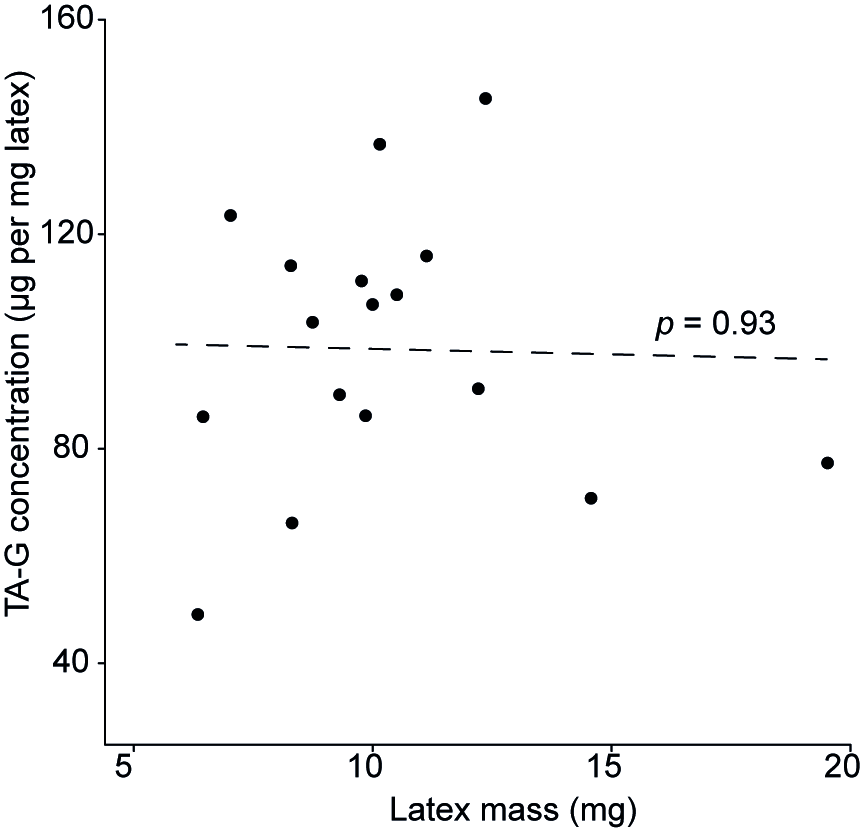

Supplement: S1 Fig — Latex fresh mass was determined by cutting the main roots 1 cm below the tiller and collecting the exuding latex. One data point represents the mean of one genotype. The p-value of a linear model is shown. Underlying data can be found in S1 Data. (TIF) [file pbio.1002332.s002.tif]

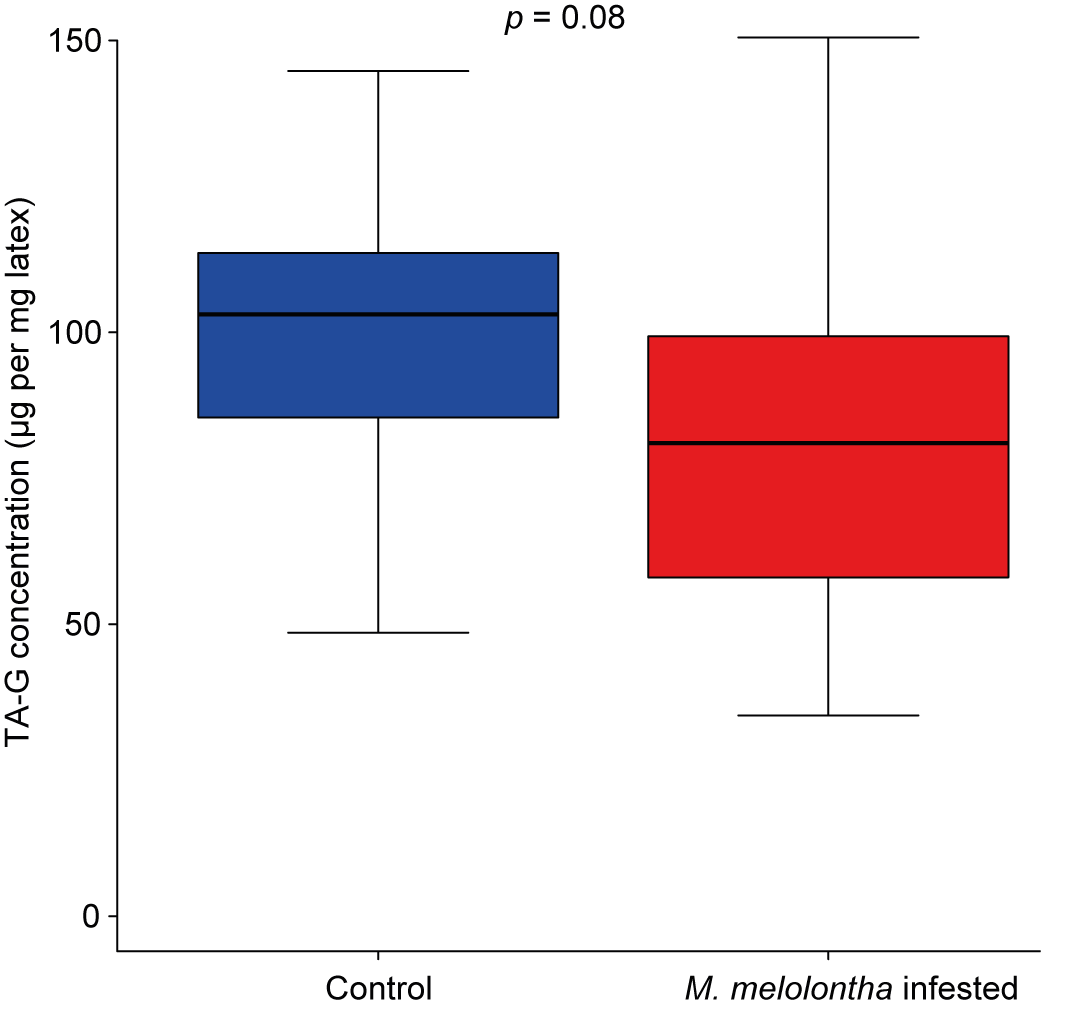

Supplement: S2 Fig — TA-G concentration was measured from 17 T. officinale genotypes with and without M. melolontha herbivory. The p-value of a t test is shown. Underlying data can be found in S1 Data. (TIF) [file pbio.1002332.s003.tif]

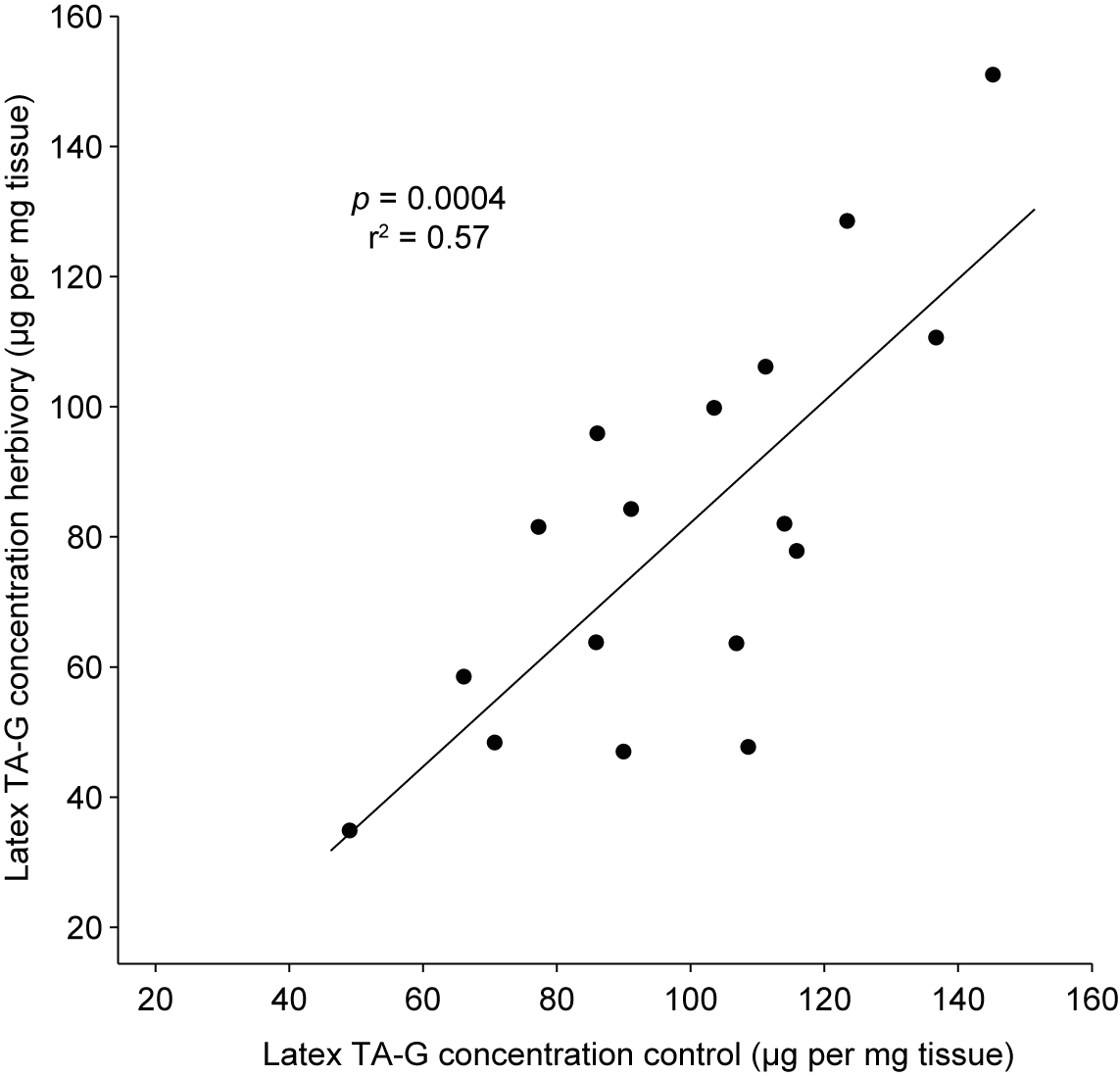

Supplement: S3 Fig — One data point represents the mean of one genotype. p-Value and r2 value of a linear model are shown. Underlying data can be found in S1 Data. (TIF) [file pbio.1002332.s004.tif]

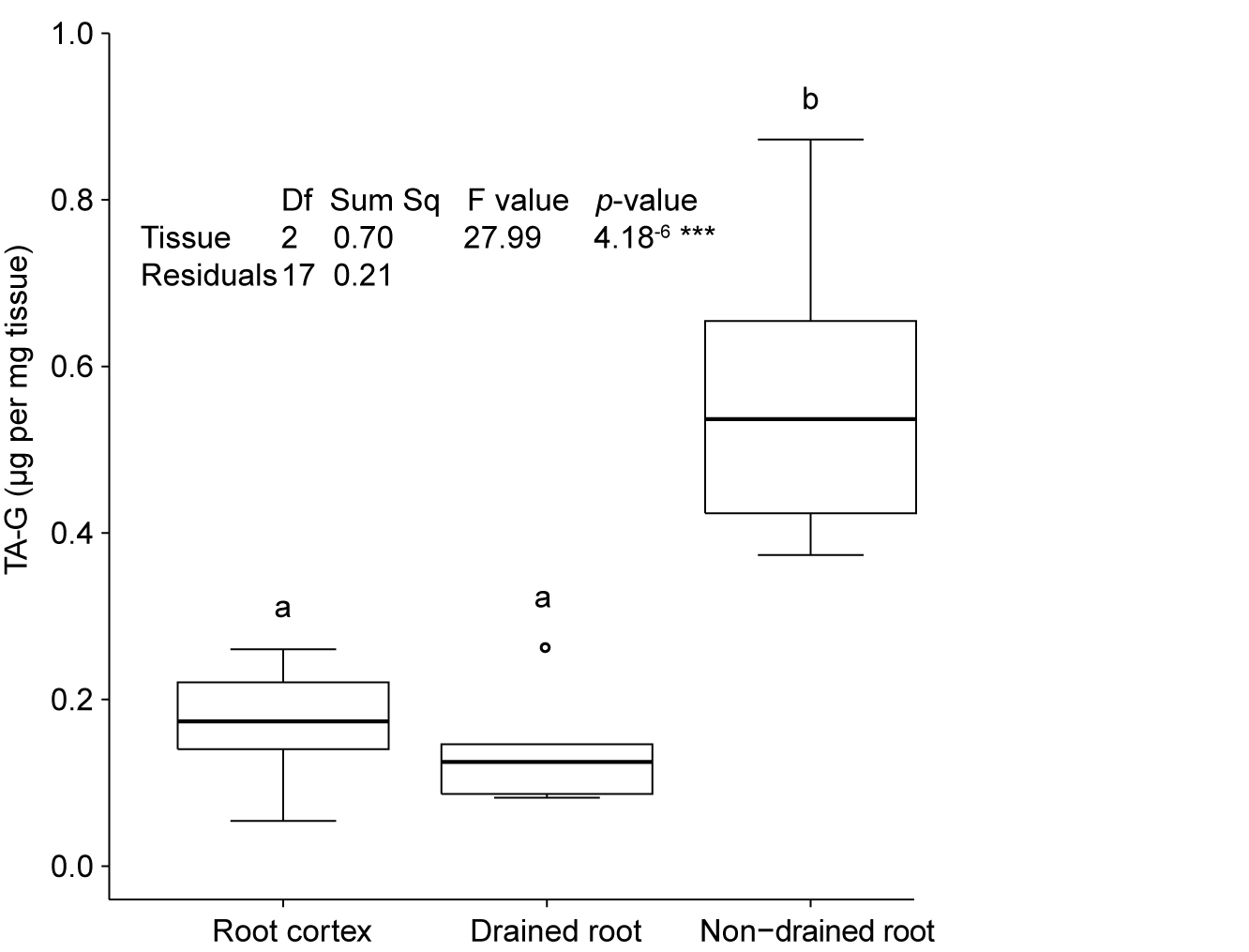

Supplement: S4 Fig — Statistics of one-way ANOVAs are shown. Different lower case letters indicate significant differences in TA-G concentrations according to Tukey posthoc tests. Underlying data can be found in S1 Data. (TIF) [file pbio.1002332.s005.tif]

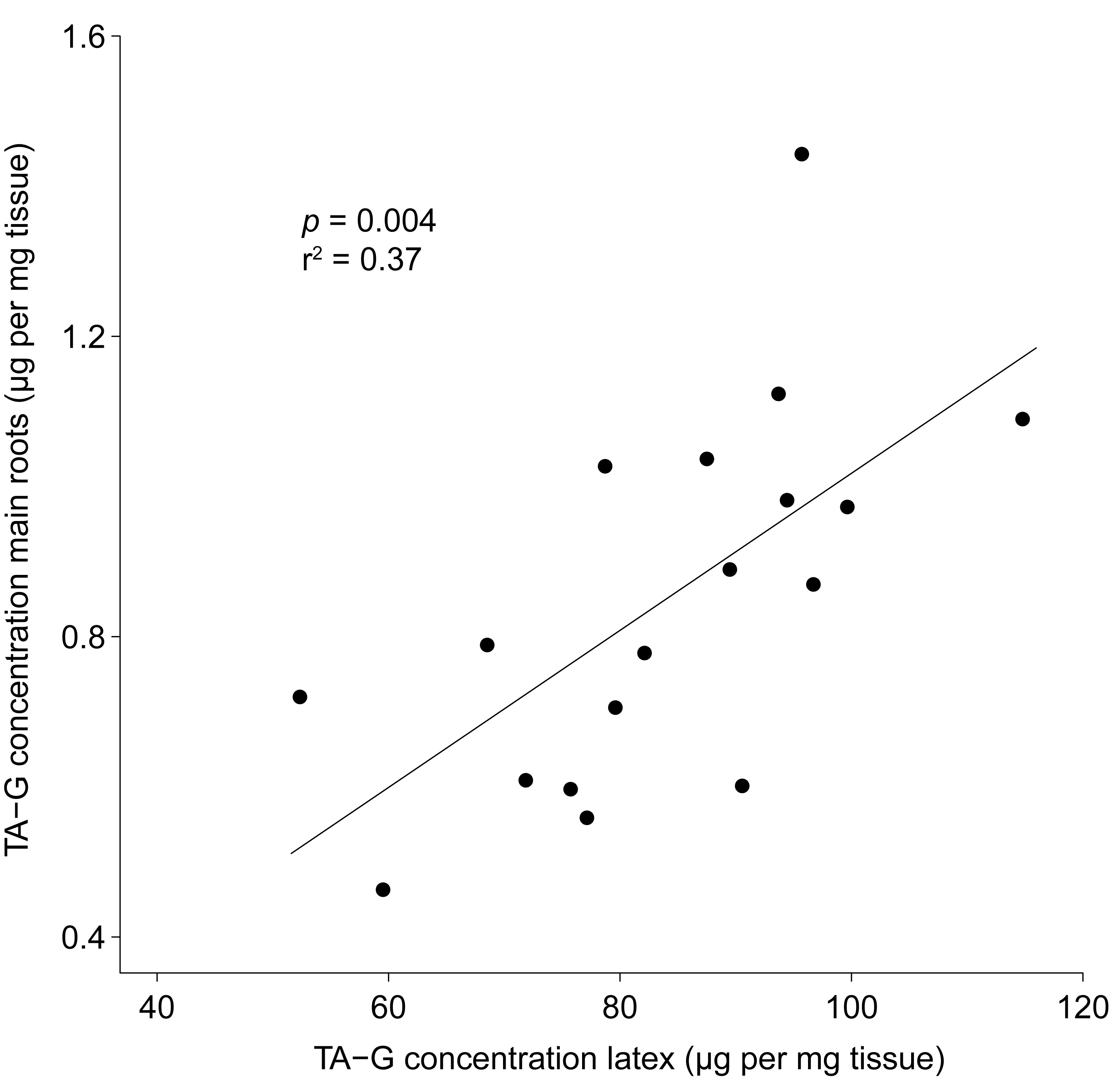

Supplement: S5 Fig — One data point represents the mean of one genotype. p-Value and r2 values of a linear model are shown. Underlying data can be found in S1 Data. (TIF) [file pbio.1002332.s006.tif]

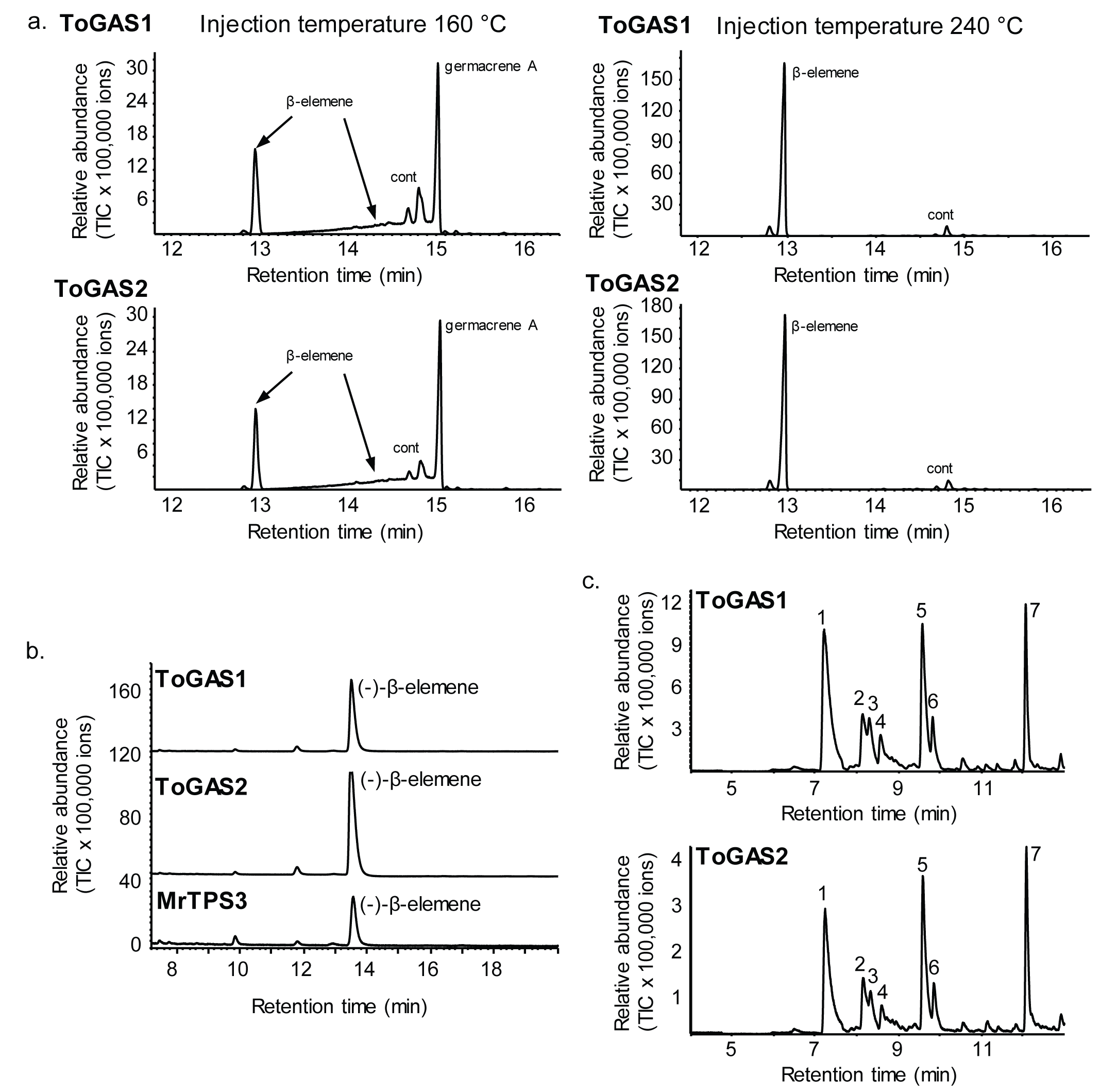

Supplement: S6 Fig — a. GC-MS analysis of enzyme products from recombinant ToGAS1 and ToGAS2 incubated with the substrate FDP. Germacrene A produced by ToGAS1 and ToGAS2 is converted to β-elemene during hot GC injection. cont, contamination. b. Chiral analysis of recombinant ToGAS1 and ToGAS2 enzyme products. Retention times and mass spectra of ToGAS enzyme products were compared to those of (-)-β-elemene obtained as a thermal rearrangement product of (+)-germacrene A synthesized by MrTPS3 from chamomile [73]. c. GC-MS analysis of monoterpene products from recombinant ToGAS1 and ToGAS2 incubated with the substrate geranyl diphosphate (GPP). 1, myrcene; 2, limonene; 3, (Z)-β-ocimene; 4, (E)-β-ocimene; 5, terpinolene; 6, linalool; 7, α-terpineol. IC = ion count. (TIF) [file pbio.1002332.s007.tif]

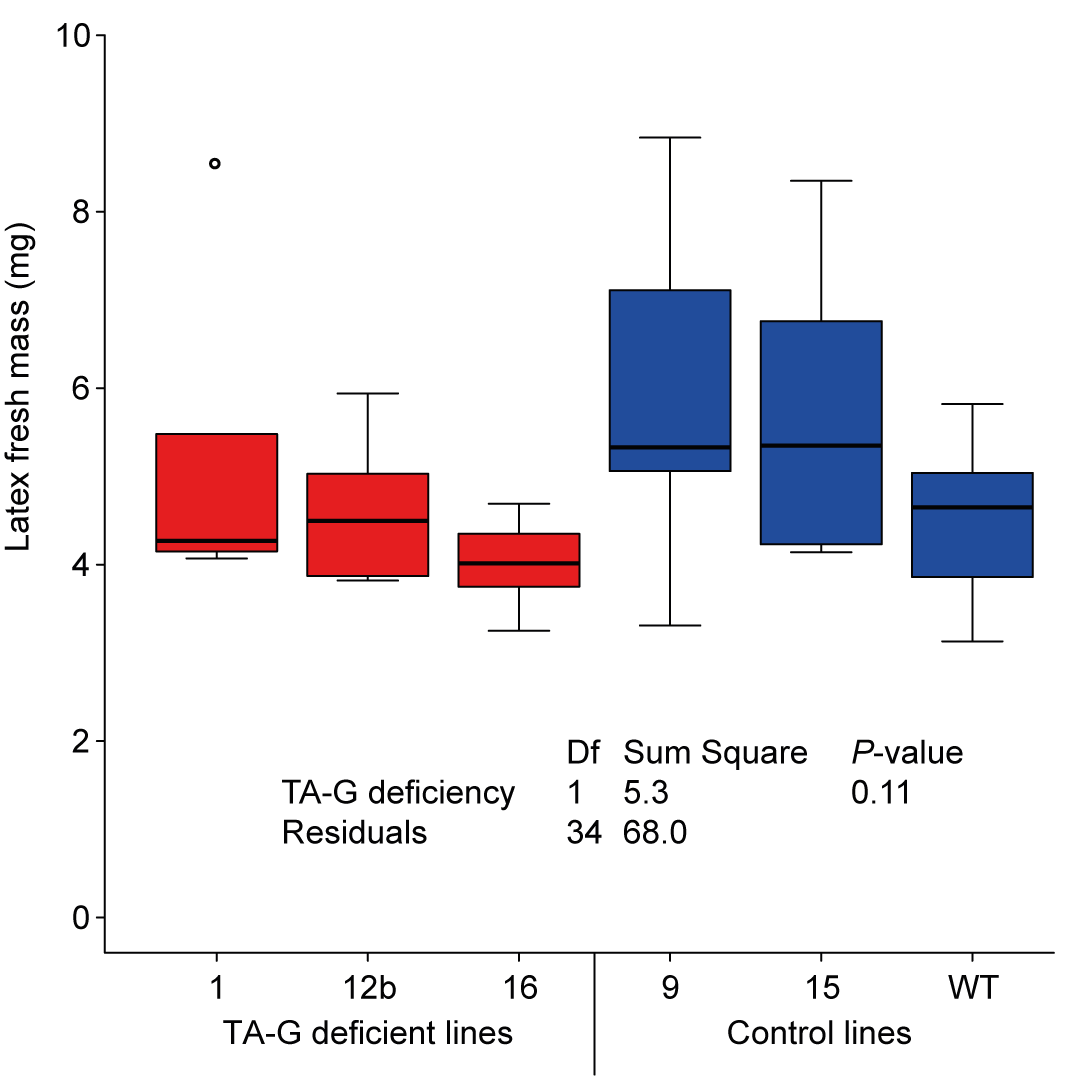

Supplement: S7 Fig — Latex fresh mass was determined by cutting the main roots 1 cm below the tiller and collecting the exuding latex. Statistics of a one-way ANOVA is shown. Underlying data can be found in S1 Data. (TIF) [file pbio.1002332.s008.tif]

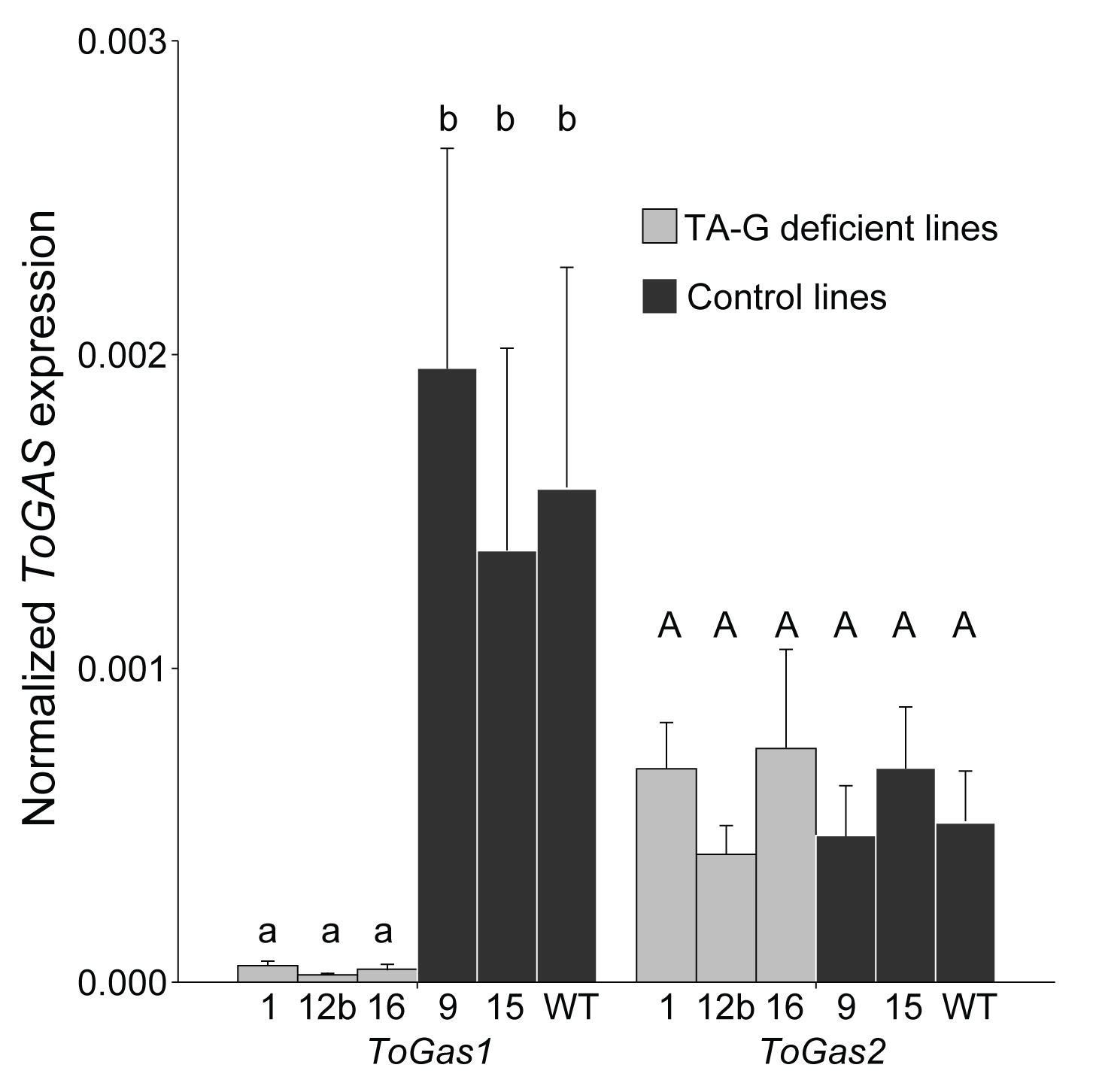

Supplement: S8 Fig — Expression of ToGAS1 and ToGAS2 of the TA-G-deficient (RNAi-1, -12b, 16) and control (RNAi-9, -15, WT) lines normalized to the elongation factor ToEF1α. Different letters indicate significant differences in expression of ToGAS1 (lower case) and ToGAS2 (upper case) between the different lines in a generalized linear model. n = 4. Underlying data can be found in S1 Data. (TIF) [file pbio.1002332.s009.tif]

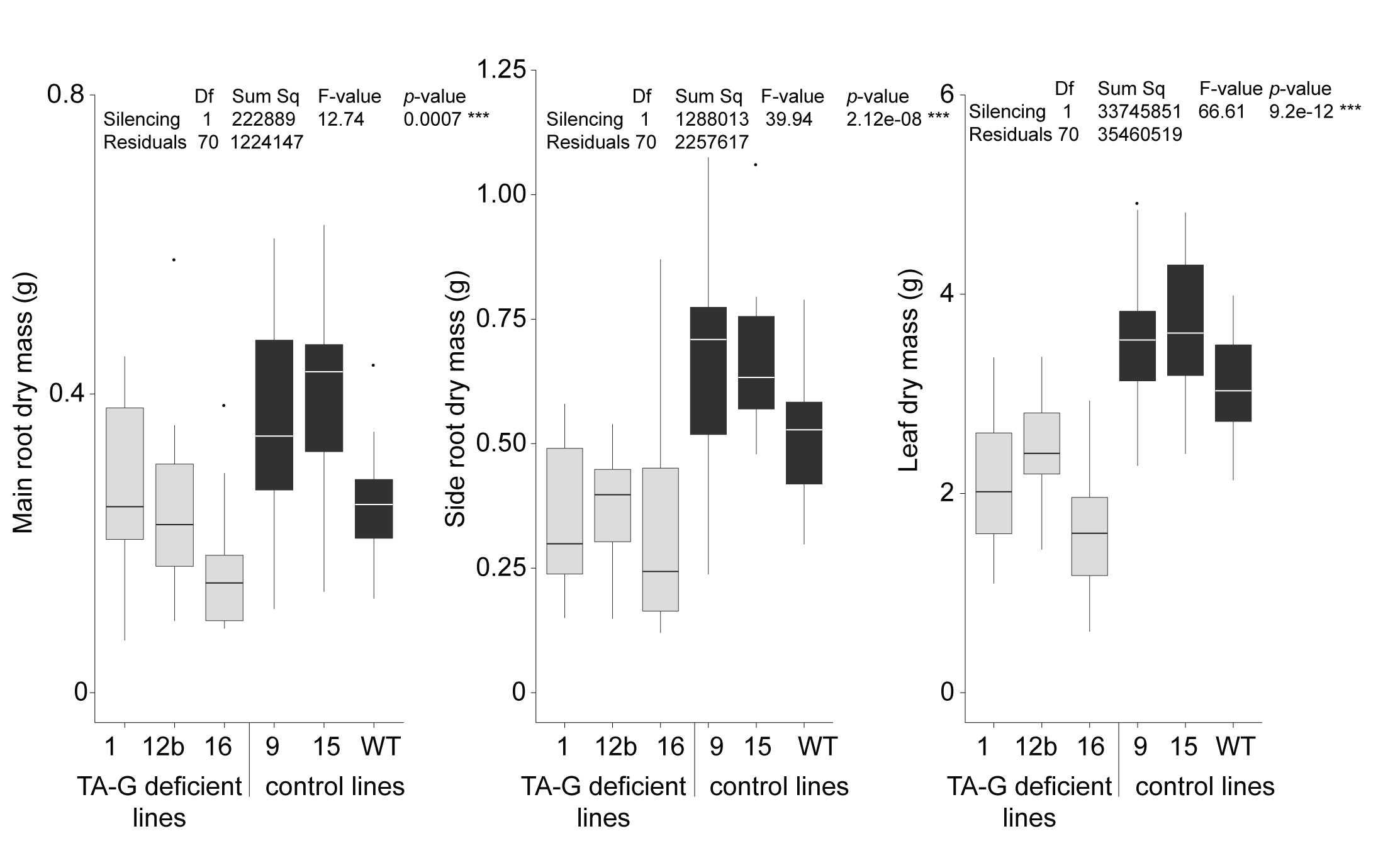

Supplement: S9 Fig — X-axis shows individual silenced lines. Statistics show one-way ANOVA. Sum Sq = sum of squares. N = 12. Underlying data can be found in S1 Data. (TIF) [file pbio.1002332.s010.tif]

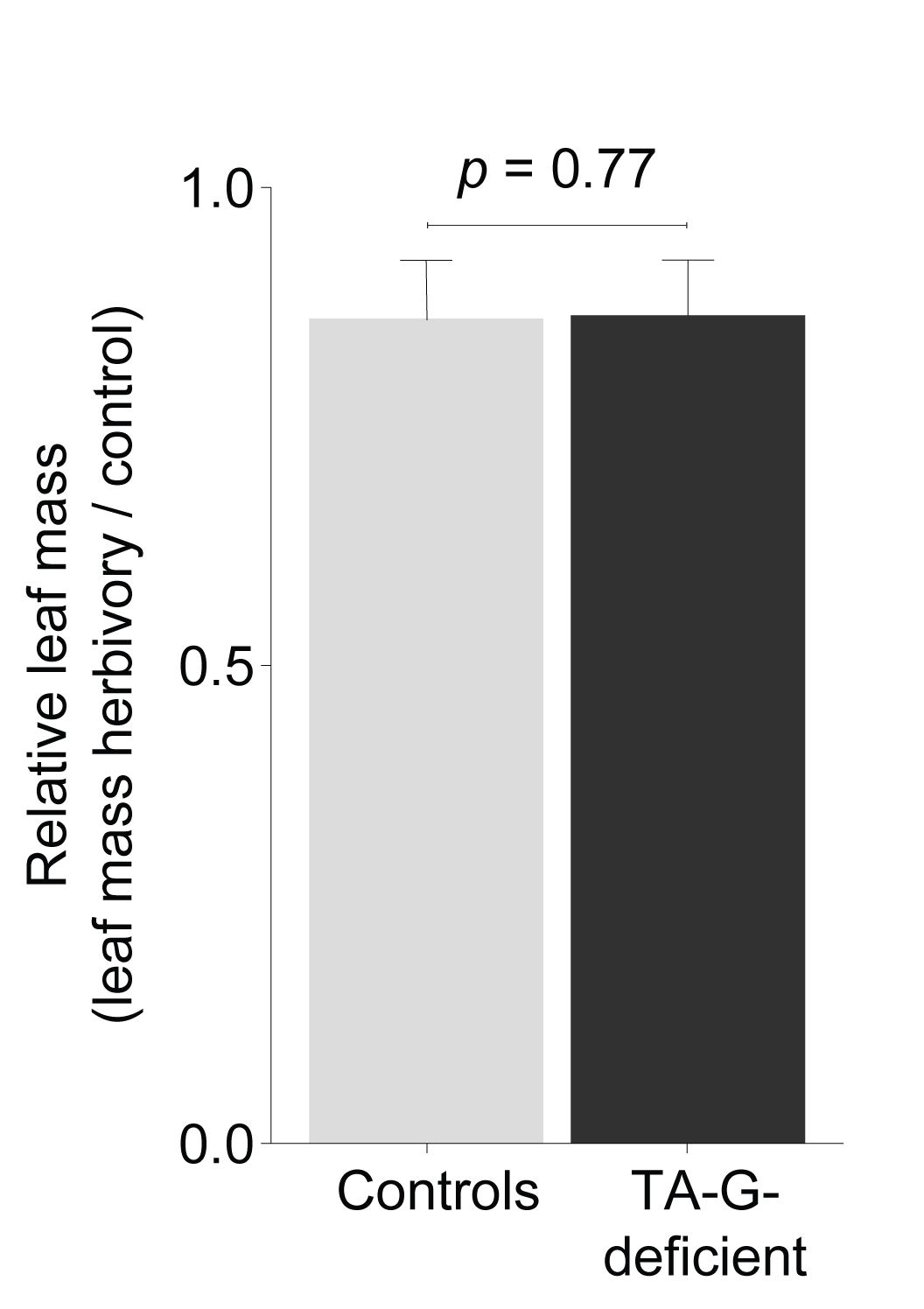

Supplement: S10 Fig — Larvae fed for 7 d on 8 wk-old T. officinale seedlings. Relative leaf mass is the mass of each herbivore infested plant relative to the mean leaf mass of the control plants of its genotype. Statistics from Kruskal-Wallis rank sum test is shown. n = 12. Underlying data can be found in S1 Data. (TIF) [file pbio.1002332.s011.tif]

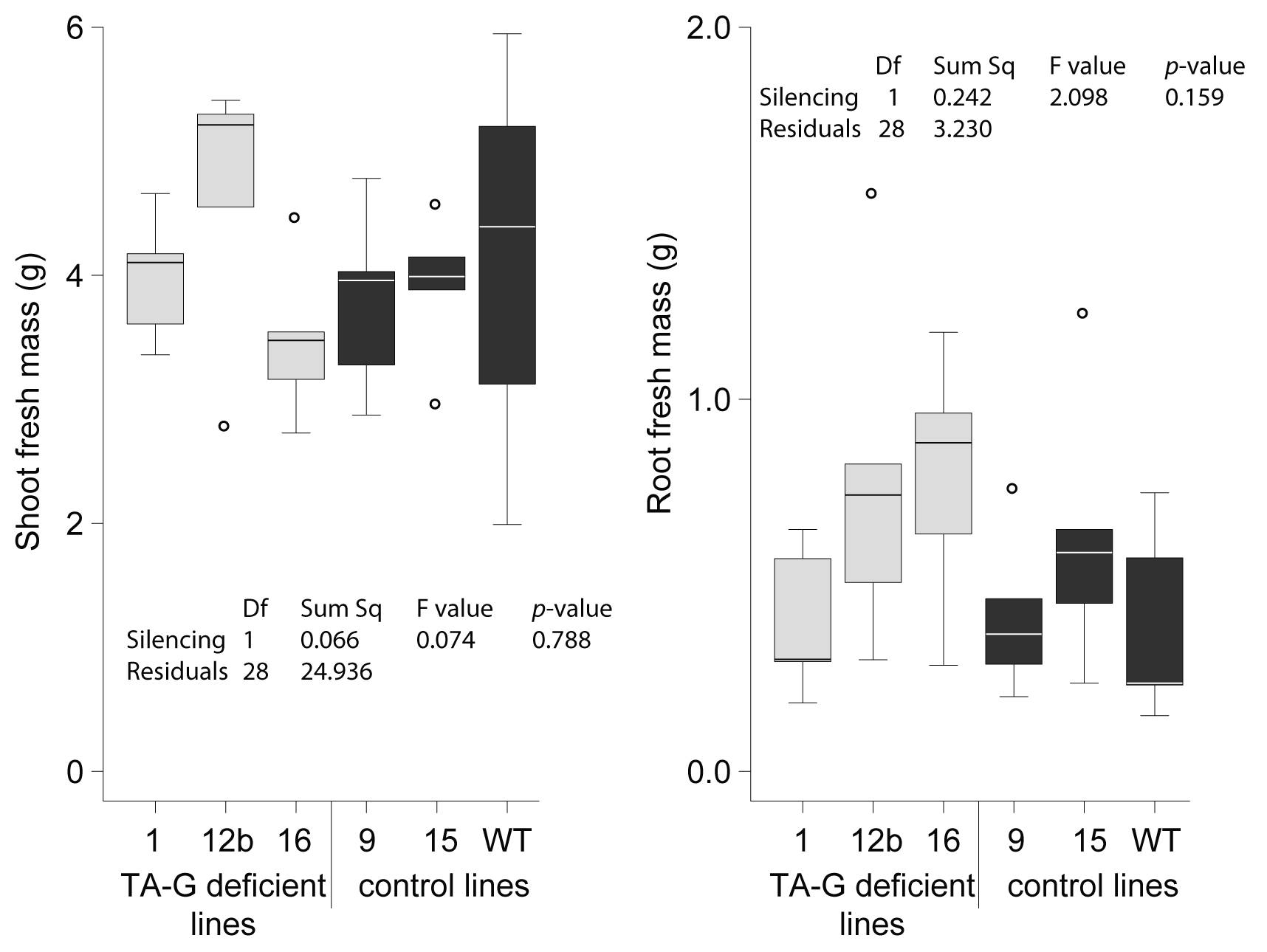

Supplement: S11 Fig — X-axis shows individual silenced lines. Statistics show one-way ANOVA. Sum Sq = sum of squares. n = 5. Underlying data can be found in S1 Data. (TIF) [file pbio.1002332.s012.tif]

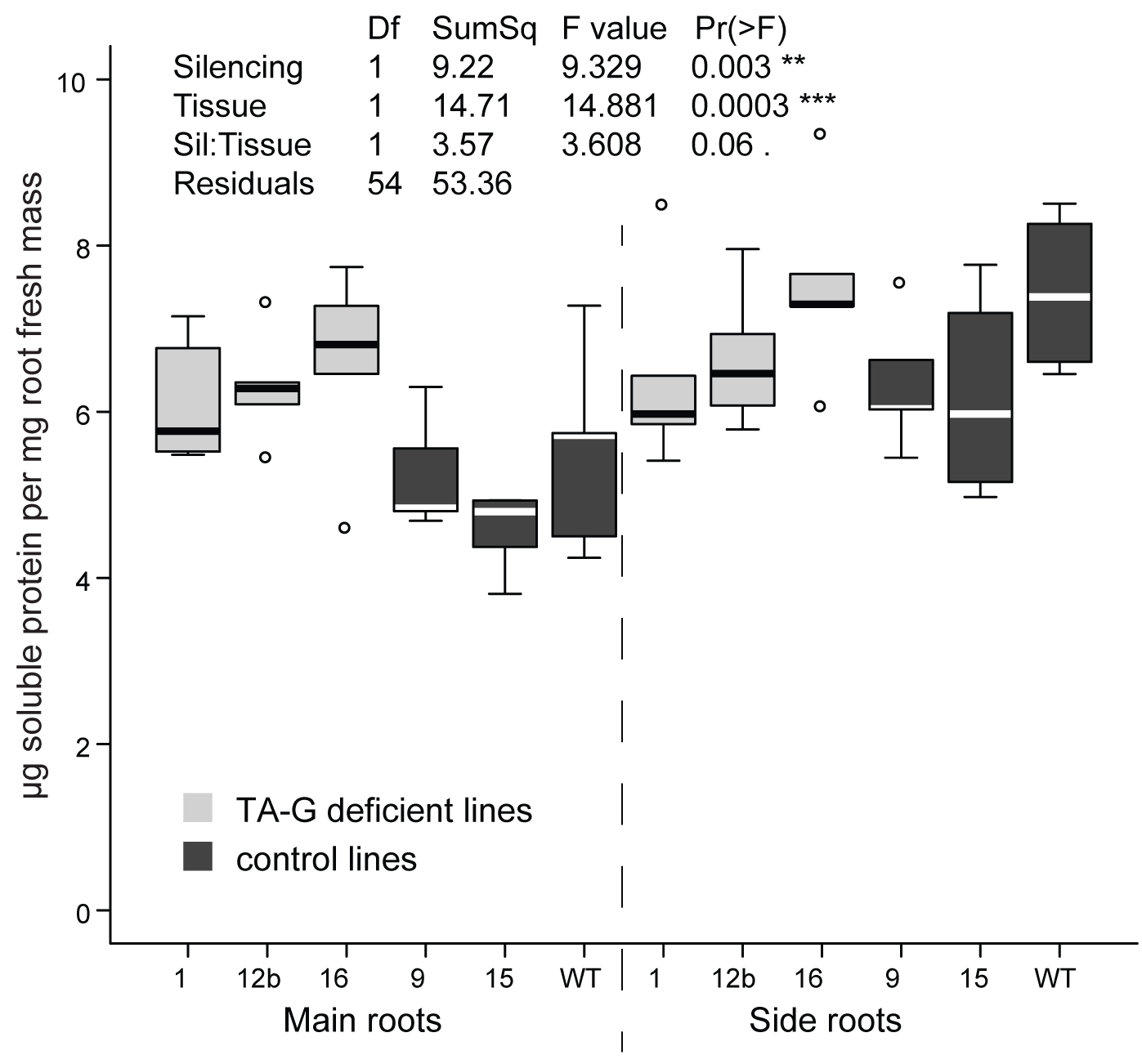

Supplement: S12 Fig — Eight week-old T. officinale were analyzed. X-axis shows individual silenced lines. Statistics of two-way ANOVA is shown. Sum Sq = sum of squares. n = 6. Underlying data can be found in S1 Data. (TIF) [file pbio.1002332.s013.tif]

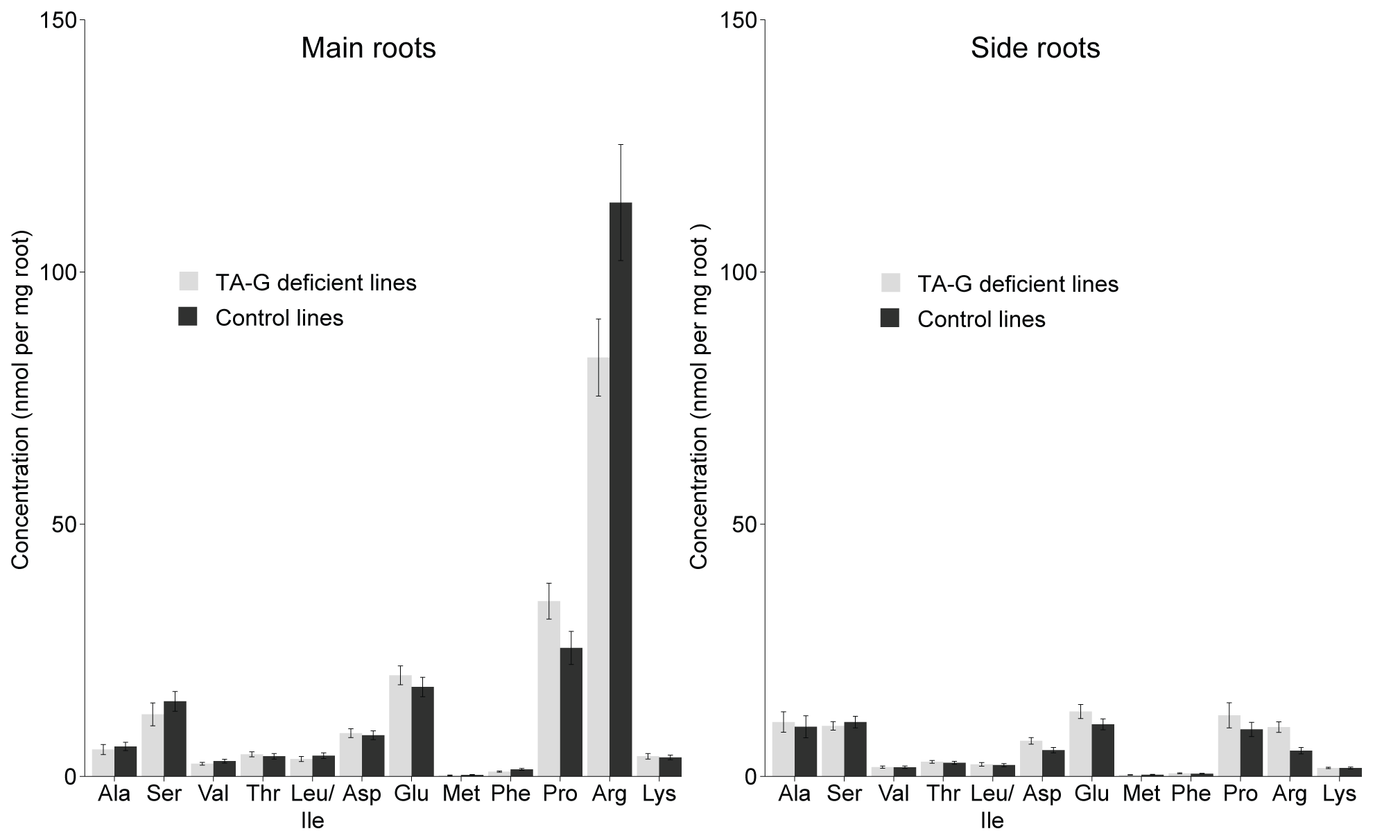

Supplement: S13 Fig — Eight week-old T. officinale were analyzed. Data from the three TA-G-deficient (RNAi-1, -12b, -16) and control lines (wild type, RNAi-9, RNAi-15) were pooled. n = 6. Underlying data can be found in S1 Data. (TIF) [file pbio.1002332.s014.tif]

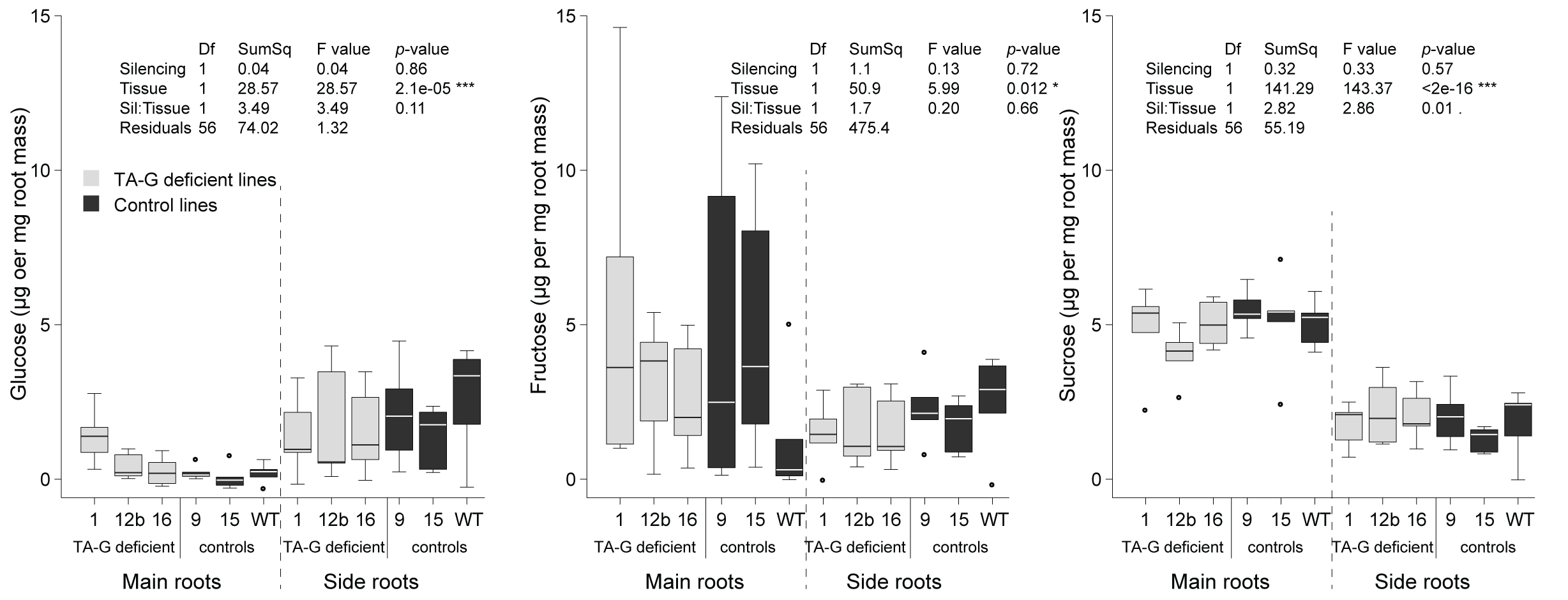

Supplement: S14 Fig — Eight week-old T. officinale were analyzed. X-axis shows individual silenced lines. Statistics of two-way ANOVA is shown. Sum Sq = sum of squares. n = 6. Underlying data can be found in S1 Data. (TIF) [file pbio.1002332.s015.tif]

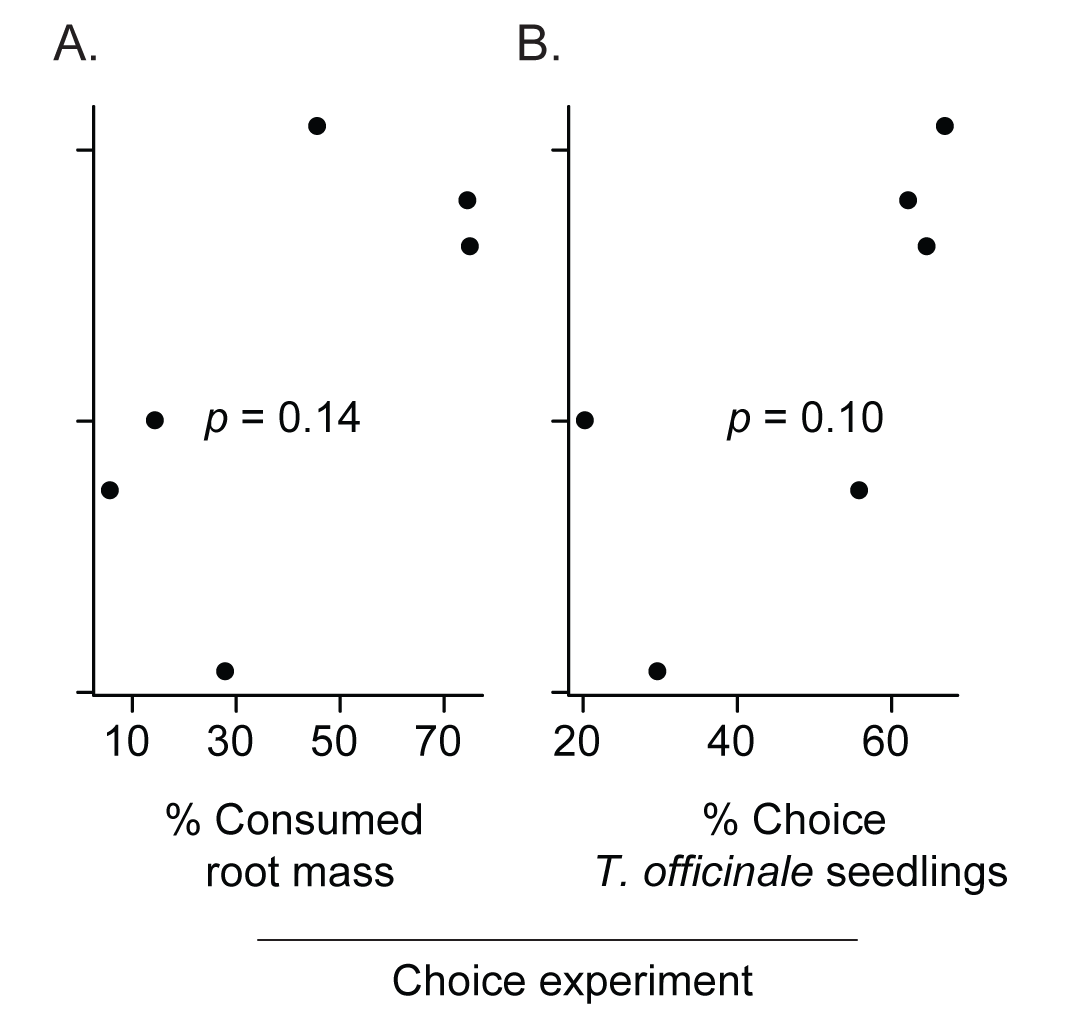

Supplement: S15 Fig — p-Values from Pearson product–moment correlations are shown. Underlying data can be found in S1 Data. (TIF) [file pbio.1002332.s016.tif]

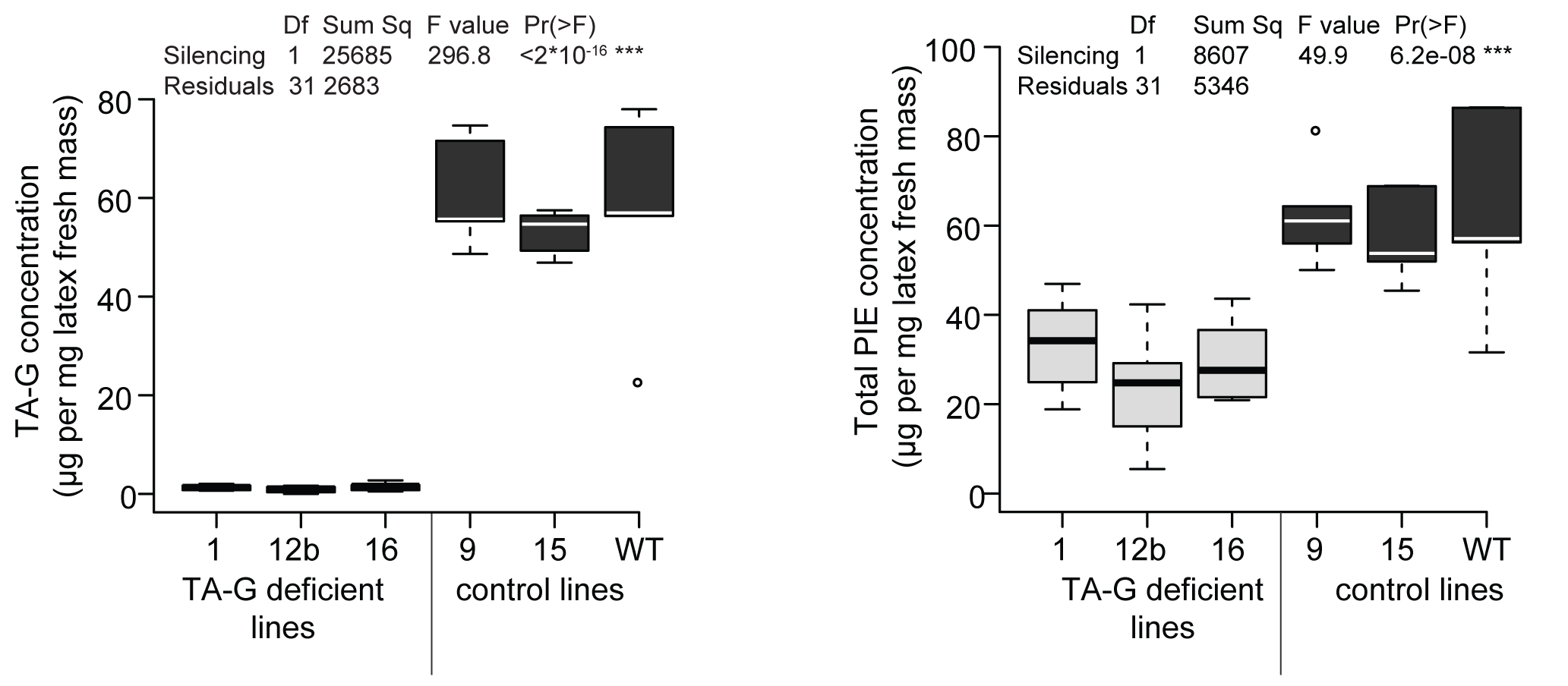

Supplement: S16 Fig — Latex of 8 wk-old T. officinale was analyzed. X-axis shows individual silenced lines. TA-G = taraxinic acid β-D-glucopyranosyl ester; PIE = phenolic inositol ester. Statistics of one-way ANOVA is shown. n = 6. Underlying data can be found in S1 Data. (TIF) [file pbio.1002332.s017.tif]

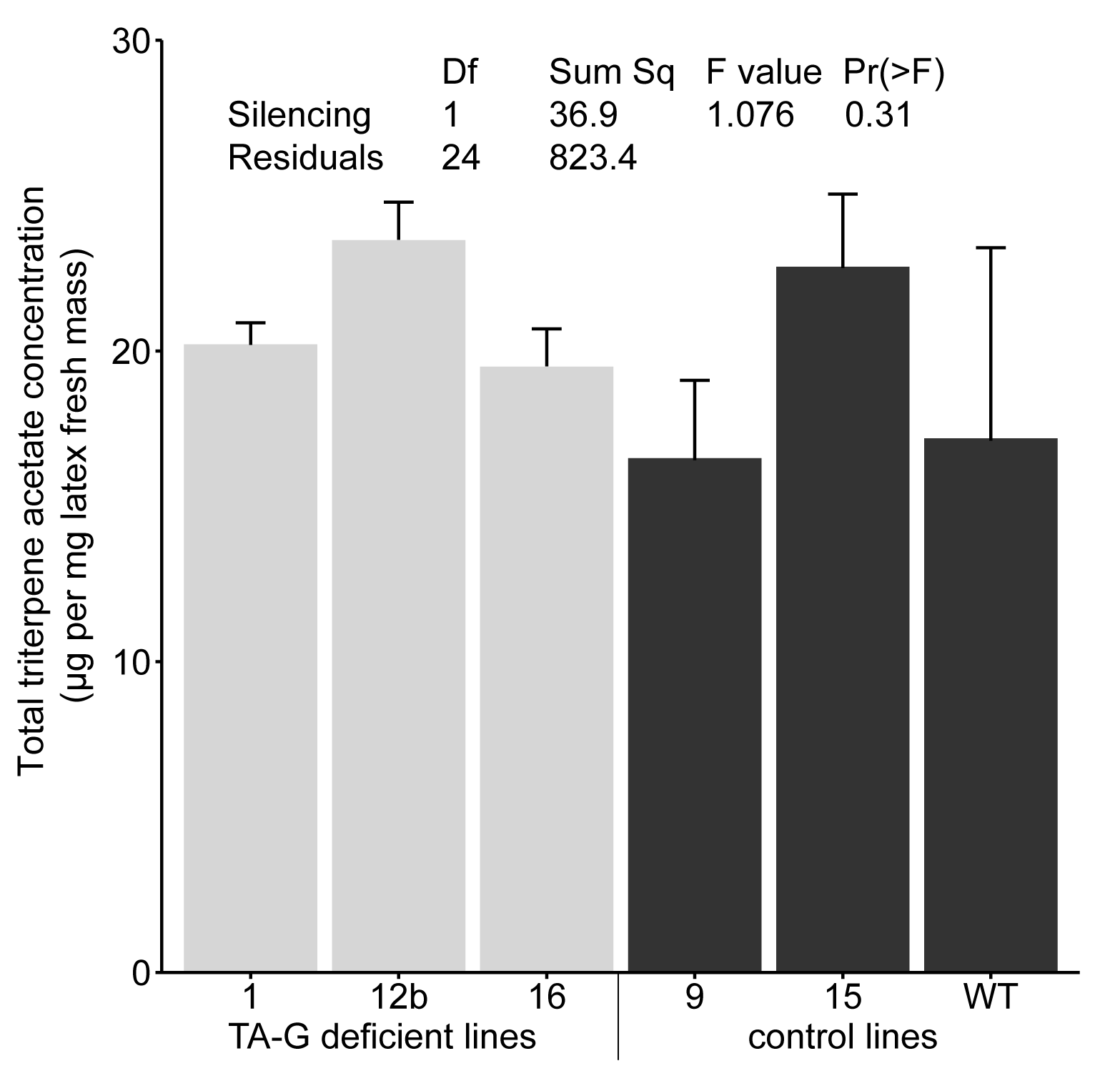

Supplement: S17 Fig — Latex of 8 wk-old T. officinale was analyzed. X-axis shows individual silenced lines. Statistics of one-way ANOVA is shown. n = 6. Underlying data can be found in S1 Data. (TIF) [file pbio.1002332.s018.tif]

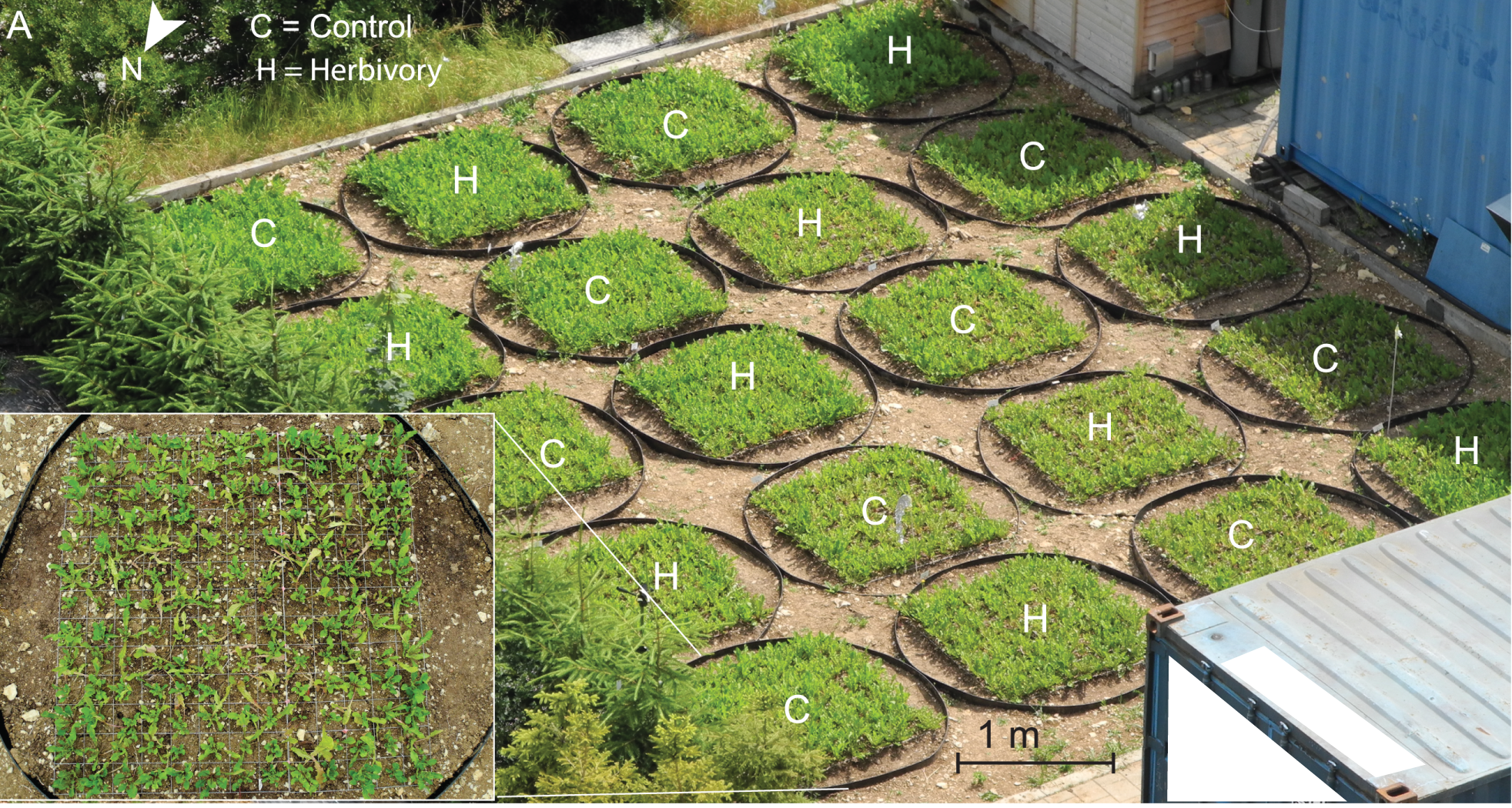

Supplement: S18 Fig — Note that container logo and number in the lower right corner have been removed during post processing of this photograph. (TIF) [file pbio.1002332.s019.tif]

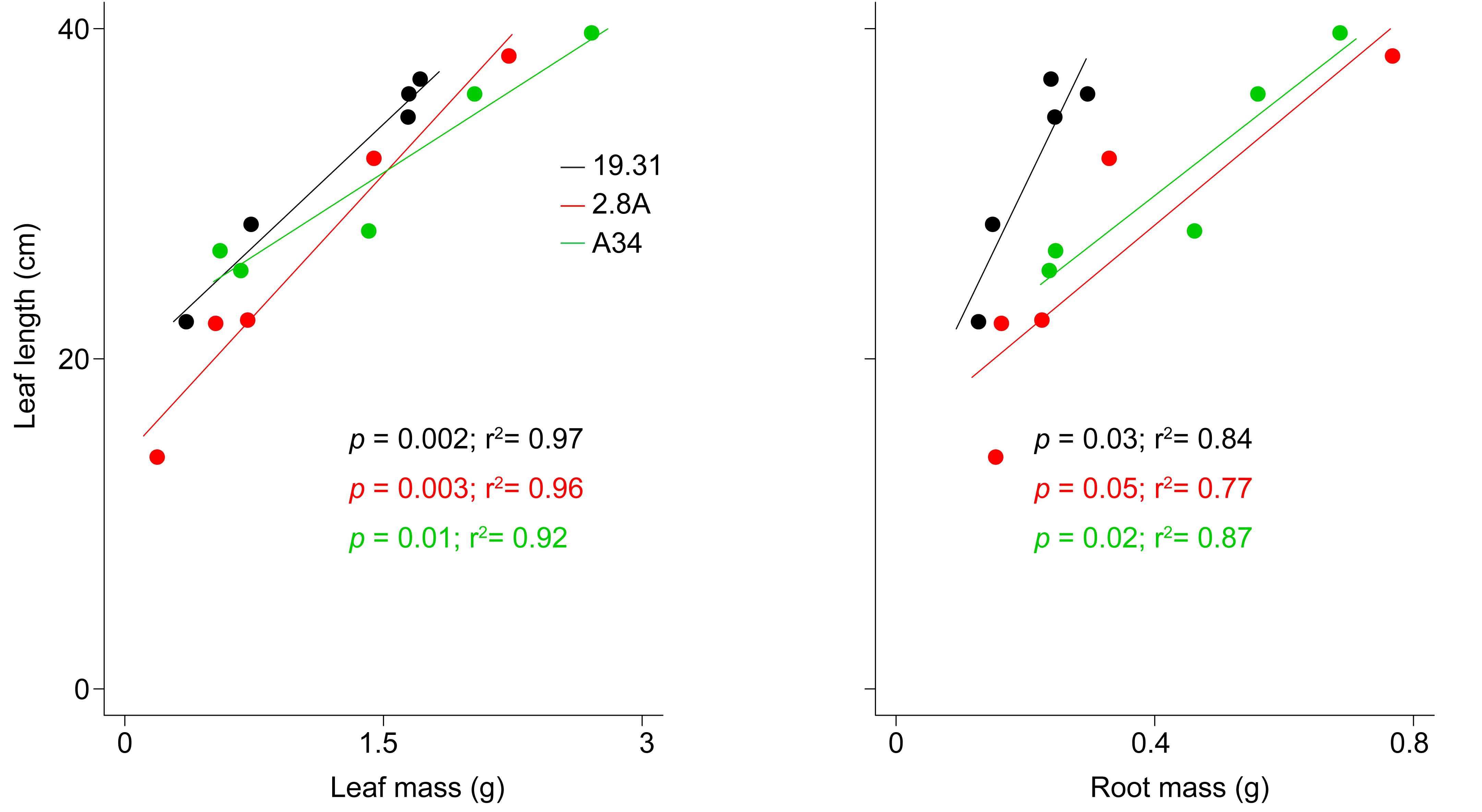

Supplement: S19 Fig — Correlation between leaf length and leaf and root dry mass across three genotypes (19.31, 2.8A, A34) over a growth period of 5 wk. Five plants per genotype were harvested every week starting with 6 wk-old plants cultivated in a growth chamber. Each data point represents the mean of each genotype and time point. Statistics from linear models are shown. Underlying data can be found in S1 Data. (TIF) [file pbio.1002332.s020.tif]

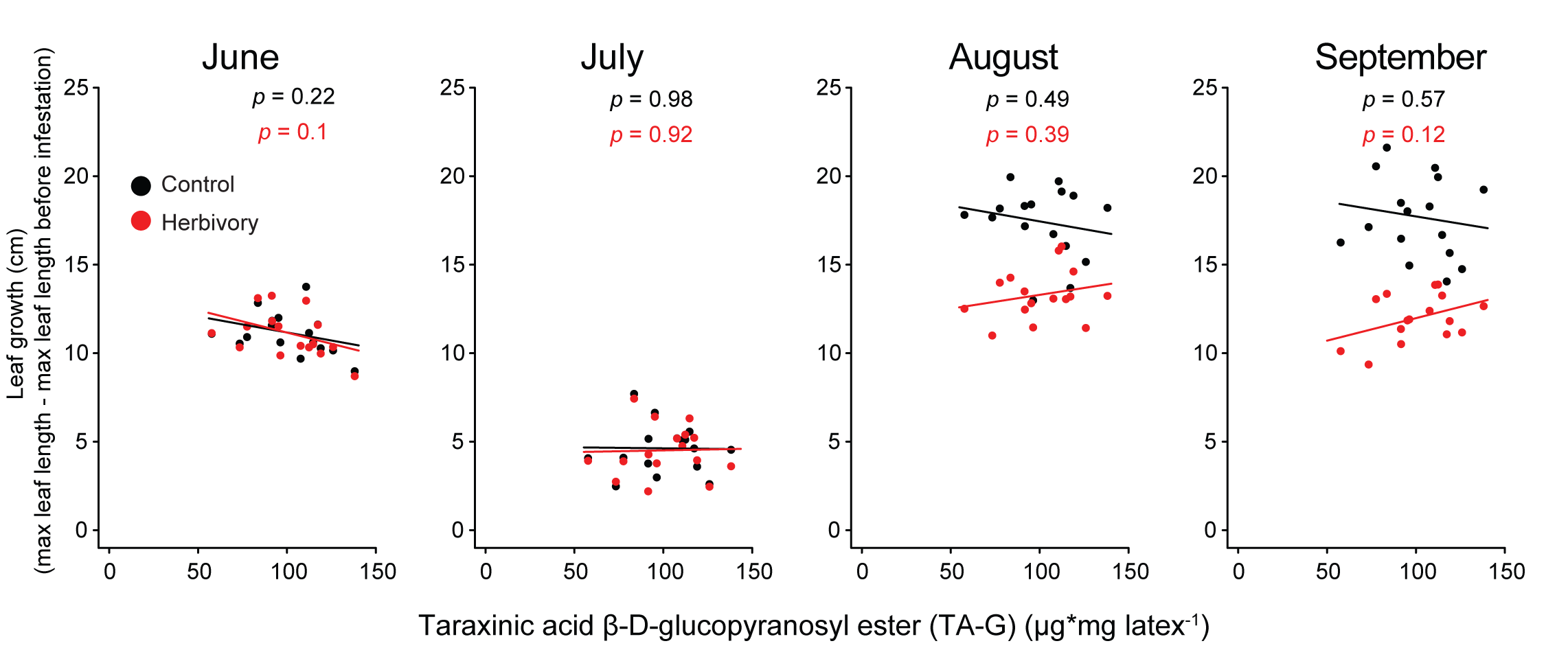

Supplement: S20 Fig — TA-G concentration tended to be positively correlated to leaf growth (maximal leaf length of each month–maximal leaf length before infestation) under M. melolontha attack and negatively correlated to leaf growth in the control treatment towards the end of the growing season. Plants were infested in June. Each data point represents the mean of one genotype. p-Values from Pearson’s product–moment correlations based on mean values of each genotype are shown. Underlying data can be found in S1 Data. (TIF) [file pbio.1002332.s021.tif]

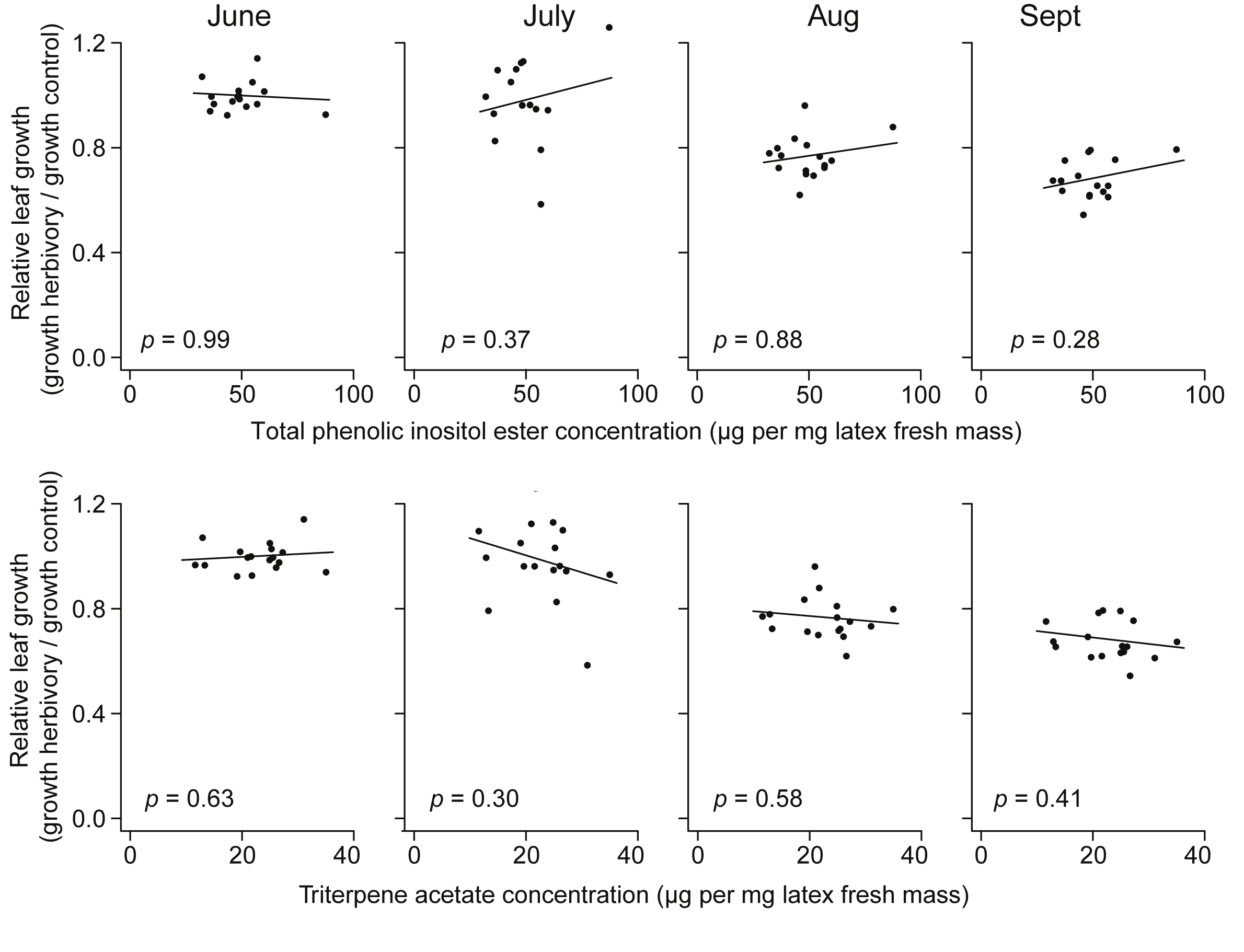

Supplement: S21 Fig — Relative leaf growth is the size increase of the longest leaf of the herbivore-infested plants compared of the size of the longest leaf before infestation, expressed relative to the leaf growth of the control plants of each genotype. Each data point represents the mean relative leaf growth of one T. officinale genotype. Plants were infested in June. p-Values from Pearson’s product–moment correlations based on mean values of each genotype are shown. Underlying data can be found in S1 Data. (TIF) [file pbio.1002332.s022.tif]

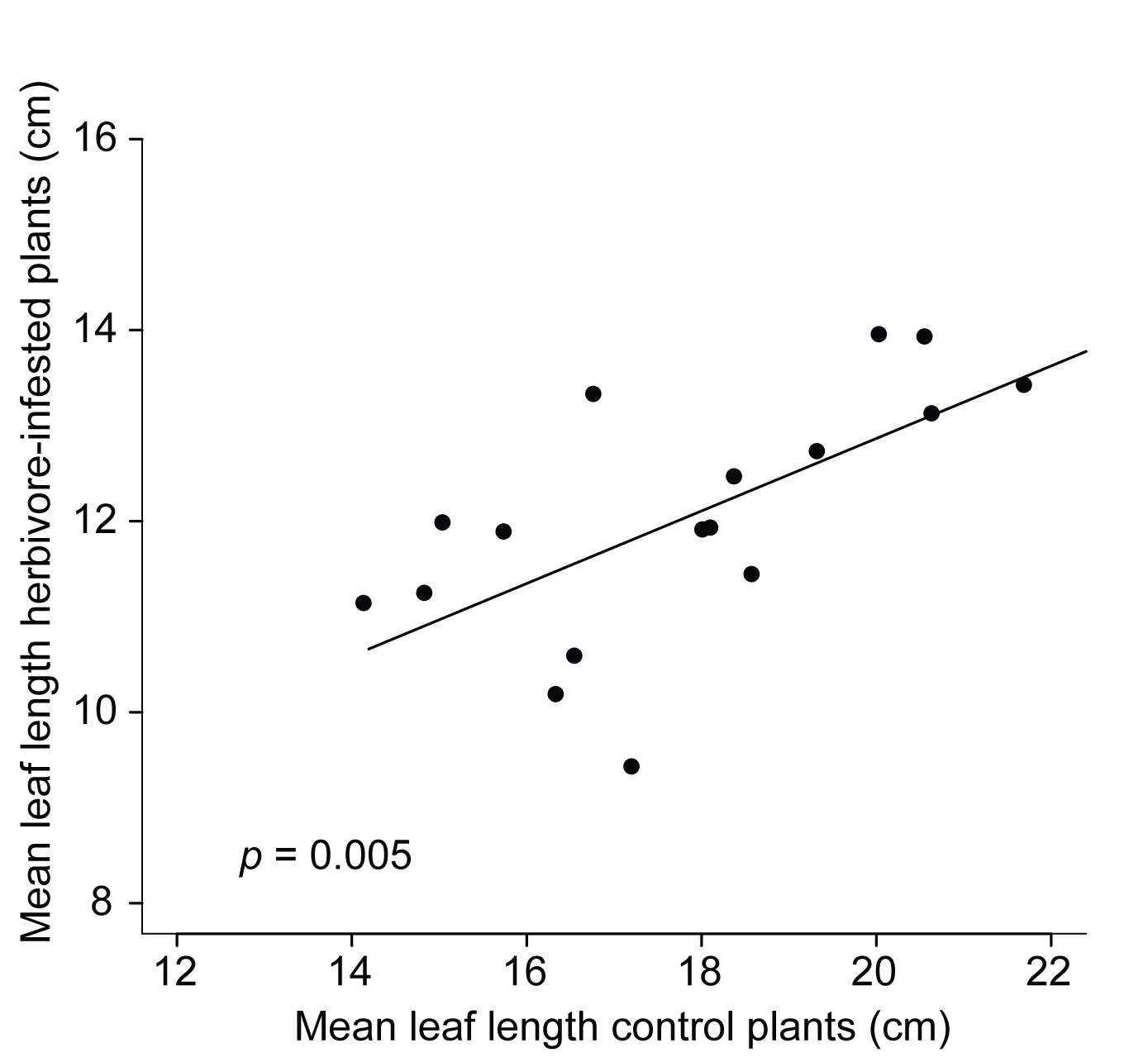

Supplement: S22 Fig — Herbivore damage was proportional to plant size. The p-value of a Pearson product–moment correlation is shown. One data point represents the mean of one genotype. Underlying data can be found in S1 Data. (TIF) [file pbio.1002332.s023.tif]

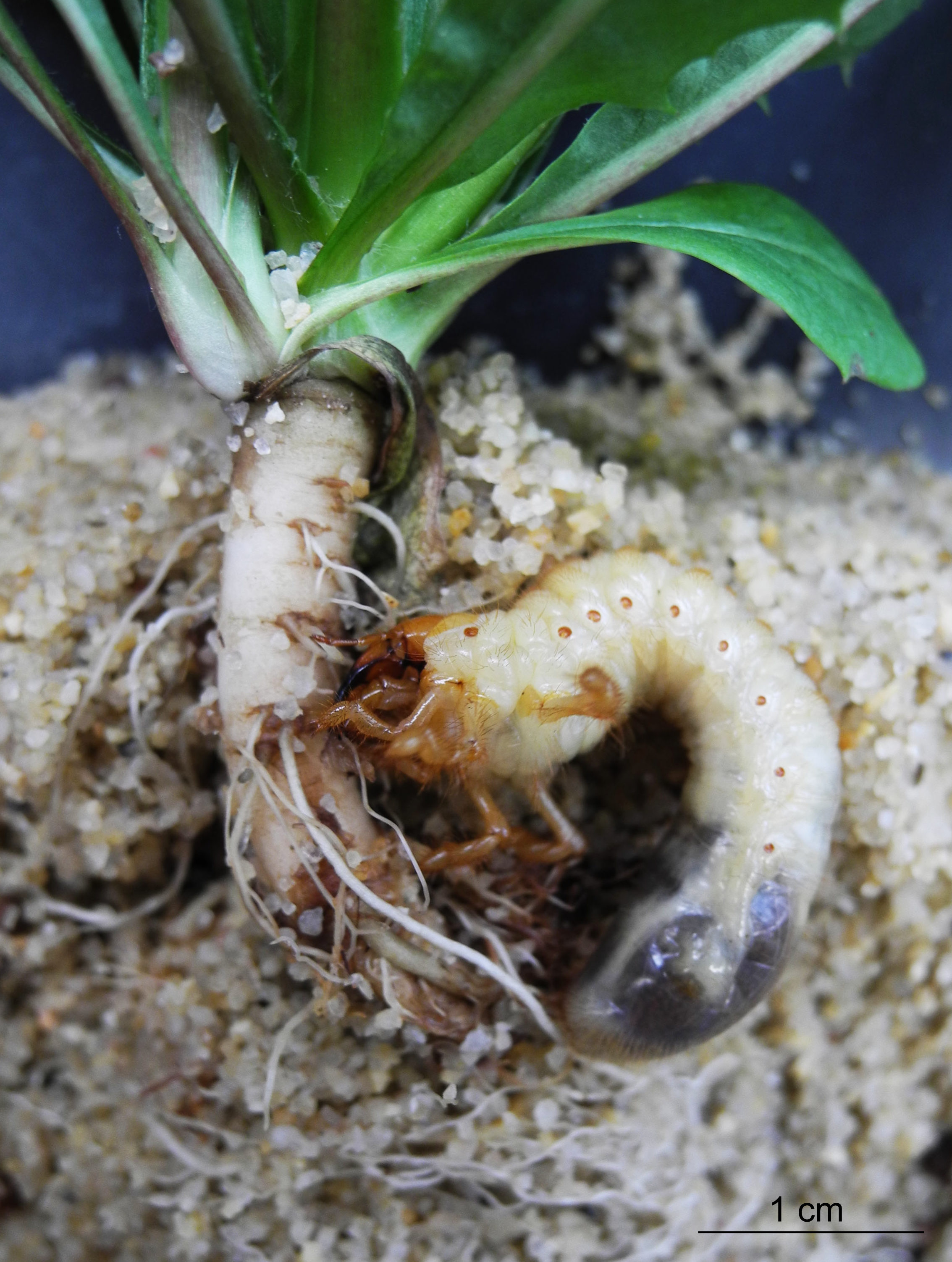

Supplement: S23 Fig — (TIF) [file pbio.1002332.s024.tif]

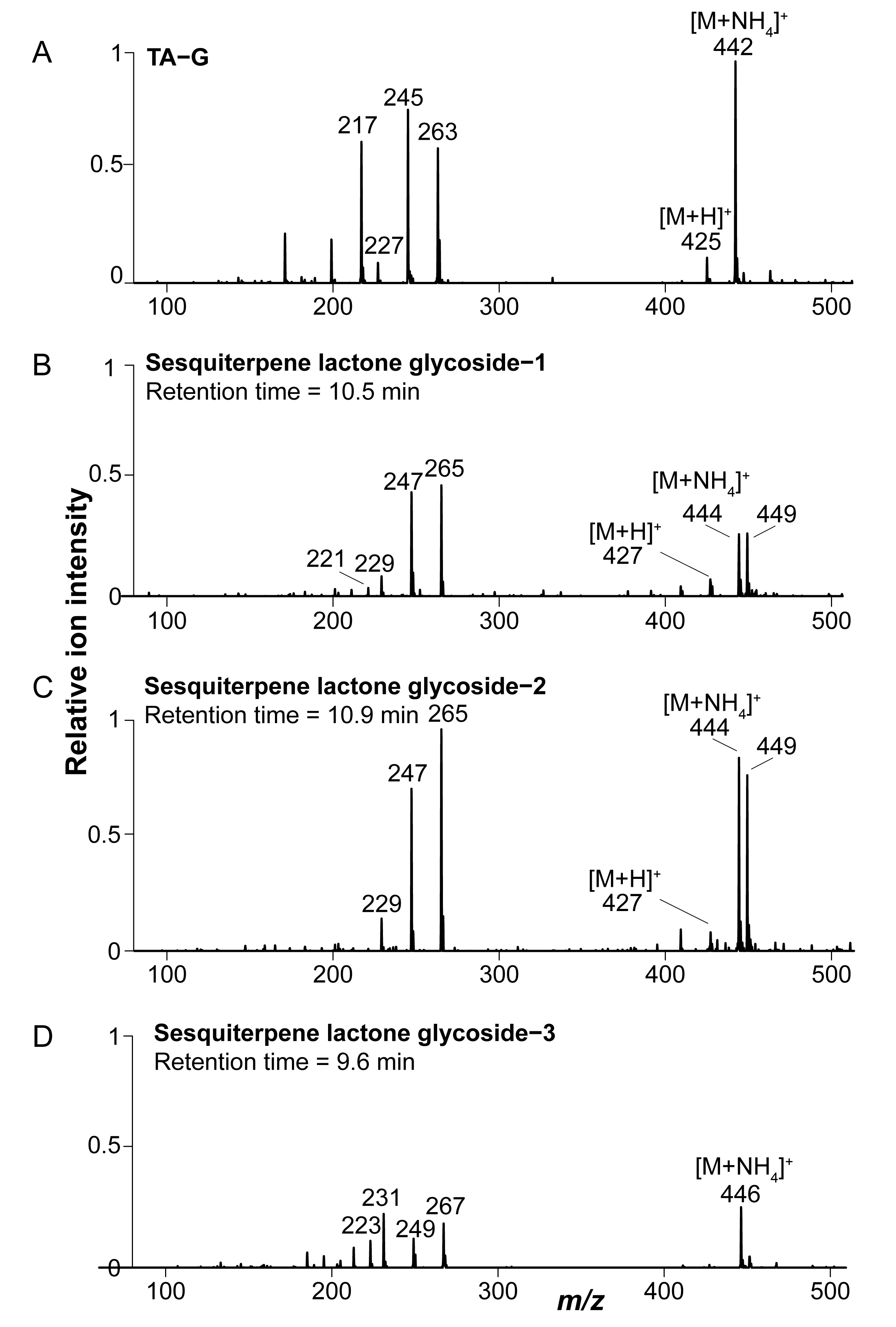

Supplement: S24 Fig — A. In-source fragmentation pattern of TA-G, obtained from a latex methanol extract of genotype A34. B–D. Putative sesquiterpene lactone glycosides. A latex methanol extract from genotype 17.20A was screened for fragmentation patterns resembling TA-G. All samples were analyzed on an Esquire 6000 ESI-Ion Trap mass spectrometer in positive ionization mode [36]. (TIF) [file pbio.1002332.s025.tif]
