# Supplementary material for: A Latex Metabolite Benefits Plant Fitness under Root Herbivore Attack
Source: PLoS Biol. 2016 Jan 5;14(1):e1002332. doi: 10.1371/journal.pbio.1002332 (PMC4701418; doi:10.1371/journal.pbio.1002332)
Supplement: S1 Table — Genotype A34 is a triploid, synthetic apomict, created by crossing a sexual diploid mother from France with diploid pollen from a triploid apomict from the Netherlands [75]. (DOCX) [file pbio.1002332.s026.docx]

| Region | Country | Longitude | Latitude | Genotypes |
| --- | --- | --- | --- | --- |
| Beroun | [Czech Republic](http://en.wikipedia.org/wiki/Czech_Republic) | 14.0891 | 49.981 | 6.56 |
| Hosingen | Luxembourg | 6.0740 | 50.0149 | 3.48 |
| Mühlheim am Main | Germany | 8.8646 | 50.1172 | 4.26 |
| Heteren | Netherlands | 5.7509 | 51.9600 | H72 |
| Wageningen-Heteren | Netherlands | 5.6627 | 51.9936 | 1.16A |
| Bockenem | Germany | 10.1088 | 52.0121 | 8.13A |
| Ostbevern | Germany | 7.8325 | 52.0328 | 2.2A; 2.8A |
| Gömnitz | Germany | 10.7297 | 54.1148 | 10.8 |
| Bentwisch | Germany | 12.2326 | 54.123 | 11.4 |
| Marum | Denmark | 12.2784 | 56.0253 | 12.56; 12.57 |
| Skaenninge | Sweden | 15.1057 | 58.3629 | 15.47 |
| Arboga | Sweden | 15.8334 | 59.4139 | 16.14, 16.17A |
| Uppsala | Sweden | 17.6123 | 59.8554 | 17.20A |
| Sikvik | Sweden | 17.2982 | 60.6657 | 18.46 |
| Soerra | Sweden | 17.0766 | 61.7083 | 19.31 |
| Haernoesand | Sweden | 17.9268 | 62.6107 | 20.3B |
| Synthetic genotype | France x Netherlands |  |  | A34 |
