# Supplementary material for: A Latex Metabolite Benefits Plant Fitness under Root Herbivore Attack
Source: PLoS Biol. 2016 Jan 5;14(1):e1002332. doi: 10.1371/journal.pbio.1002332 (PMC4701418; doi:10.1371/journal.pbio.1002332)
Supplement: S2 Table — (DOCX) [file pbio.1002332.s027.docx]

|  | Estimate | Standard error | *P*-value |
| --- | --- | --- | --- |
| TA-G concentration | -0.0015 | 0.0004 | 0.003 ** |
| Latex mass | 0.008 | 0.0034 | 0.030 * |
