# Supplementary material for: A Latex Metabolite Benefits Plant Fitness under Root Herbivore Attack
Source: PLoS Biol. 2016 Jan 5;14(1):e1002332. doi: 10.1371/journal.pbio.1002332 (PMC4701418; doi:10.1371/journal.pbio.1002332)
Supplement: S3 Table — (DOCX) [file pbio.1002332.s028.docx]

|  | Estimate | Std. Error | *P*-value |
| --- | --- | --- | --- |
| Total TA-G | -2.6^-5^ | 4.5^-5^ | 0.56 |
