# Supplementary material for: A Latex Metabolite Benefits Plant Fitness under Root Herbivore Attack
Source: PLoS Biol. 2016 Jan 5;14(1):e1002332. doi: 10.1371/journal.pbio.1002332 (PMC4701418; doi:10.1371/journal.pbio.1002332)
Supplement: S4 Table — (DOCX) [file pbio.1002332.s029.docx]

| **name** | **species** | **accession number** |
| --- | --- | --- |
| 8-*epi*-cedrol synthase 1  8-*epi*-cedrol synthase 2  (*E*)-β-caryophyllene synthase  amorpha-4,11-diene synthase 1  amorpha-4,11-diene synthase 2  amorpha-4,11-diene synthase 3  (*E*)-β-farnesene synthase  germacrene A synthase  (-)-β-pinene synthase  (-)-(*3R*)-linalool synthase 1  (-)-(*3R*)-linalool synthase 2  germacrene A synthase 1  germacrene A synthase 2  germacrene A synthase 1  germacrene A synthase 2  germacrene A synthase 3  δ-cadinene synthase  germacrene A synthase  germacrene A synthase 1  germacrene A synthase 2  (*E*)-β-caryophyllene synthase  α-isocomene synthase  germacrene A synthase  ocimene synthase  germacrene D synthase  (+)-germacrene D synthase  (-)-germacrene D synthase  (+)-germacrene A synthase  kaurene synthase A | *Artemisia annua*  *Artemisia annua*  *Artemisia annua*  *Artemisia annua*  *Artemisia annua*  *Artemisia annua*  *Artemisia annua*  *Artemisia annua*  *Artemisia annua*  *Artemisia annua*  *Artemsia annua*  *Cichorium intybus*  *Cichorium intybus*  *Heliantus annuus*  *Heliantus annuus*  *Heliantus annuus*  *Heliantus annuus*  *Ixeris dentata*  *Lactuca sativa*  *Lactuca sativa*  *Matricaria recutita*  *Matricaria recutita*  *Matricaria recutita*  *Matricaria recutita*  *Matricaria recutita*  *Solidago canadensis*  *Solidago canadensis*  *Solidago canadensis*  *Zea mays* | AAF80333  CAC08805  AAL79181  CAB94691  AAF61439  AAF98444  AAX39387  ABE03980  AAK58723  AAF13357  AAF13356  AAM21658  AAM21659  ACA14463  ACA33925  ACZ50512  ACA33926  AAL92481  AAM11626  AAM11627  AFM43734  AFM43735  AFM43736  AFM43737  AFM43738  AAR31144  AAR31145  CAC36896  AAA73960 |
