# Supplementary material for: A Latex Metabolite Benefits Plant Fitness under Root Herbivore Attack
Source: PLoS Biol. 2016 Jan 5;14(1):e1002332. doi: 10.1371/journal.pbio.1002332 (PMC4701418; doi:10.1371/journal.pbio.1002332)
Supplement: S5 Table — Leaf growth is the increase in maximal leaf length compared to maximal leaf length before infestation. (DOCX) [file pbio.1002332.s030.docx]

| Month | Latex mass | Total TA-G |
| --- | --- | --- |
| June | 0.29 | 0.79 |
| July | 0.43 | 0.51 |
| August | 0.90 | 0.38 |
| September | 0.44 | 0.49 |
