# Supplementary material for: A Latex Metabolite Benefits Plant Fitness under Root Herbivore Attack
Source: PLoS Biol. 2016 Jan 5;14(1):e1002332. doi: 10.1371/journal.pbio.1002332 (PMC4701418; doi:10.1371/journal.pbio.1002332)
Supplement: S6 Table — Relative leaf growth is the mean leaf growth of herbivore-infested plants of each genotype during the infestation period compared to the mean leaf growth of the control plants of each genotype (leaf growth: increase in maximal leaf length compared to maximal leaf length before infestation). Std. Error = Standard error. (DOCX) [file pbio.1002332.s031.docx]

| **Date** | **Estimate** |  | **Std. Error** |  | ***P*-value** |  |
| --- | --- | --- | --- | --- | --- | --- |
|  | *TA-G* | *Latex* | *TA-G* | *Latex* | *TA-G* | *Latex* |
| June | -0.0005 | 0.004 | 0.0007 | 0.005 | 0.48 | 0.37 |
| July | -0.0001 | -0.01 | 0.002 | 0.013 | 0.97 | 0.45 |
| August | 0.002 | 0.001 | 0.001 | 0.006 | 0.09 | 0.84 |
| September | 0.002 | -0.002 | 0.0008 | 0.005 | **0.02** | 0.68 |
