# Supplementary material for: A Latex Metabolite Benefits Plant Fitness under Root Herbivore Attack
Source: PLoS Biol. 2016 Jan 5;14(1):e1002332. doi: 10.1371/journal.pbio.1002332 (PMC4701418; doi:10.1371/journal.pbio.1002332)
Supplement: S7 Table — Initial density of M. melolontha in the herbivory treatment was 23 M. melolontha larvae per m2. (DOCX) [file pbio.1002332.s032.docx]

| Treatment | *M. melolontha* density per m^2^  ± standard error |
| --- | --- |
| Control | 0 ± 0.0 |
| Herbivory | 0.86 ± 0.27 |
