# Supplementary material for: A Latex Metabolite Benefits Plant Fitness under Root Herbivore Attack
Source: PLoS Biol. 2016 Jan 5;14(1):e1002332. doi: 10.1371/journal.pbio.1002332 (PMC4701418; doi:10.1371/journal.pbio.1002332)
Supplement: S1 Text — (DOCX) [file pbio.1002332.s033.docx]

To select 20 triploid dandelion genotypes that maximally vary in the latex chemical defenses, we screened 40 genotypes coming from a transect ranging from Czech Republic to Sweden in the greenhouse. 12 individuals of each genotype were cultivated in sand in a growth chamber for eight weeks in three temporally separated batches. During harvest, main root was cut 0.5 cm below the tiller, exuding latex collected into Eppendorf tubes and immediately frozen in liquid nitrogen. To measure coagulation rate, main root was cut once more and a 2 µl capillary was hold for 90 s into the exuding latex before recording the height of the latex inside the capillary. Leaf mass was dried for two days at 60 °C and weighed. Latex was extracted and analyzed on HPLC-DAD as described previously, and peak area of taraxinic acid β-D-glucopyranosyl ester and of phenolic inositol esters integrated at 275 nm. To select genotypes that maximally differed in the latex defenses, all genotypes that significantly differed in above ground biomass according to a Tukey post hoc test were excluded to reduce the effects of plant growth on plant resistance. Next, hierarchical clustering with Eucledian distance and Ward linkage was performed based on following latex chemical traits: concentration of taraxinic acid β-D-glucopyranosyl ester and major phenolic inositol esters, latex mass and coagulation rate. The dendrogram was then cut into 20 clusters and the most frequent genotype of each cluster was chosen. Data analysis was performed in R (1) using the package agricolae (2).

References

1. R Core Team. A language and environment for statistical computing. Vienna, Austria: R Foundation for Statistical Computing; 2014.

2. Mendiburu Fd. agricolae: Statistical Procedures for Agricultural Research. R package version 1.2-0 ed2014.
