# Supplementary material for: A Latex Metabolite Benefits Plant Fitness under Root Herbivore Attack
Source: PLoS Biol. 2016 Jan 5;14(1):e1002332. doi: 10.1371/journal.pbio.1002332 (PMC4701418; doi:10.1371/journal.pbio.1002332)
Supplement: S2 Text — (DOCX) [file pbio.1002332.s034.docx]

**ToGAS1**

ATGGCAGCAGTTGAAGCCAATGGGACCCTGCAACCAAACACCAAAACCACCATAGAGCCAGTGCGCCCTTTGGCAAATTTCCCTCCTTCAGTATGGGGTGATCGTTTCCTGTCATTCTCTCTTGACAATACGGAATTGGAAGGATATGCAAAAGCTATGGAGGAGCCCAAAGAAGTAGTGAGAAGATTGATCGTAGATCCAACCATGGATTCCAATAAGAAACTAAGTTTGATATACTCTGTACACCGTCTTGGTTTGACTTATATGTTCTTGCAAGATGTTGAAGCGCAGCTTGACAAACTTTTCAAAGAGTTTAACTTGCAAGATTATGAAGAAGTTGATCTATACACAACTTCCATCAACTTTCAAGTTTTCCGACACCTTGGTCACAAACTGCCTTGTGATGTATTTAACAAATTCAAGGACAGTAGCTCGGGTACATTCAAGGAGTGTGTTACCAAGGATGTGAAGGGTATGTTAGGATTATATGAATCTGCCCAATTGAGATTAAGAGGAGAAGAGATTCTAGATGAAGCCGCGGAATTCACTGTAACTCAaCTCAAGAGTGTAGTAAACACTTTAGAAGGCAAGCTTGGACAACAGGTGATGCAATCATTGAGGAGACcATTTCATCAAGGGATGCCAATGGTGGAGGCAAGGTTTTATTTCGCCaACTATGATGAAgAATGCTCCACACATGAGTCGCTaACAAAGCTTGCAAAGTTGCATTTcAACTATTTACAGCTACaACAAAaGGAAgAACTTCGCATTGtCTcaAAgtgGtGGAAGGATATGAGGTTCCAGGAAACTACTCCTTATATAAGGGATAGAGTACCAGAGATTTACTTGTGGATATTGGGATTATACTTTGAGCCTCGTTACTCTTtGGCACGAATCATCGCCACAAAAATTACATTGTTTCTTGTGGTGCTAGATGACACATATGATGCTTATGCTACTATTGAAGAAATTCGCCTTCTAACAGATGCCATAAATAGGTGGGACATTAGTGCCATGGAACAACTTCCAGAATACATTCGACCATTCTTCAAAATTCTCCTAGACGAGTATGCTGAACTTGAGAAACAACTCGCTAAAGAAGGAAGAGAAAAAAGTGTTTTTGCTTCAAAAGAAGCGTTCCAAGACATTGCTAATGGCTACCTTGAAGAGGCCGAGTGGACAAACAGTGGATATGTTGCATCTTTTCCTGAATACATGAAGAATGGTTTAATCACTTCTGCCTACAATGTTATTTCCAAATCTGCTTTAGTGGGTATGGGGGAGATGGTTGGTGAAGATGCCTTGGCTTGGTATGAAAGTCATCCAAAGACATTGCAAGCTTCAGAGTTAATTTCAAGACTCCAAGATGATGTCATGACTTACCAGTTTGAACGAGAAAGGGGACAATCAGCCACCGGCGTGGATTCTTATATCAAGACTTATGGGGTGTCAGAAAAGGAAGCTATTAACGAGCTCAATAAAATGATTGAAAACGCCTGGAAAGACATAAACGAAGGCTGCCTTAAGCCAAGAGAAGTGTCTATGGATTTGCTTGCTCCAATTGTTAATCTTGCACGAATGATAGATGTGGTATACAGGTACGACGATGGGTTCACTTTTCCGGGAAAGACCATGAAAGAGTATATTACTCTATTGTTTGTAGGTtCTTCGCCCATGTAA

**ToGAS2**

ATGGCTCTAGTTAGAAACAACAGTAGCAATGGCCGTGAACCAGTGCTCAGTCCTAGGAGCCTCACTAGCCCCCGTGGGTTAACCAGCCCCCGACCATTGGCAGCCCGACCAACTCCGGAGCCGGTTCGCCCTTTGGCCAACTTTCCACCTTCCATATGGGCAGATCGATTCATCACATTCTCTCTTGATAACTCAGAATTGGAAGCTTACGCAAATGCACTCGAAGAGCCAAAAGAAGCAGTGAGGAGTTTGATAACTGATACTACCATTGATGCAACCACAAAATTAAAGTTGATCTACTCAGTGCACCGTCTCGGTTTATCATATCTTTATCCAGAAGAGATTGATGCTGAGCTTGACATACTCTTCAAGAAAATTGACTTAGATTATTACGAACAAGTTGATTTGTACACCATTTCAGTCCAATTTCAAGTTTTCAGACACCATGGTTACAGATTATCTTCTGATATATTTAAAAAGTTTAAGGATACTACTACGGGTATATTCACGGACGAAGTCTCAAAAGACGTGAAAGGCATGTTGAGTTTATATGAGTCGGCACACTTGAGGCTACATGGTGAAGACATCTTAGATGAAGCTTTGGCATTCACTGAAGCTCAACTCAAAAAGATCGTGAGCACACTTGAGGGAGATCTTGCACGCCAAGTGAATCAAGTATTAAAGAGACCTTTCCACACTGGAATGCCAATGGTGGAGGCAAGACTATATTTTAACACACACGAAGAAGACTTTTCGTGCCATGAAGCGGTTGTAAAGCTAGCTAAAATCAACTTCAACTATTTGCAACTACAACAAAAGGAAGAACTTCGTATGGTTTCACAGTGGTGGAAAGATATGGAGTTTCAAACATCGGTCCCTTACATAAGAGATAGAGTACCAGAGATATACCTATGGATTTTGGGGTTATACTTCGAGCCATATTACTCTCGGGCACGTATCATAGCAACAAAAATCACATTGTTCTTGGTGGTTTTAGATGACACATATGACGCATACGCTACTATTGATGAGATCCGATTGATCACAGACGCTATTAATAGGTGGGAAATGAGCGCGATAGATCAACTTCCCCAATATATCAAACCATTCTTCCGAATTCTCCTAAACGAGTATGATGATCTCGAGAAAGAATACTCTAAGGATGGAAGAGCTTTCAGTGTCCATGCTTCAAAACAAGCATTTCAAGAAATAGCACGAGGGTATCTTGAAGAGGCGGAGTGGTTAAACAATAATTATGTGGCGACATTTCCCGAGTATATGAGGAATGGATTGATTACTTCAGCTTATAATGTCATTTCAAAATCAGCACTTGTGGGTATGGGTGCAATTGCAGATGAAGAGGCTCTTGCTTGGTTTGAAACACATCCCAAAATTTTAAAAGCTTCGGAGTTGATTTCAAGACTACAAGACGATGTTATGACTTTTCAGTTTGAGAGGAAACGAGGACAGTCGGCTACAGGTGTGGATGCTTATATCAAGGAGTACAAAGTATCTGAGGAAGTGGCAATCAAAGAGCTCATGAAAATAATTGAAAACGCATGGAAAGATATAAACGAAGGATGCCTAAAACCTACCGAAGTCTCGATGGCACTACTAACTCCTATCTTAAATCTTGCACGAATGATAGATGTGGTATACAAATTTGATGATGGGTTCACTTTTCCTGGGAAAACCTTAAAAGACTATATTACCCTTTTGTTCGTTAGTCCTCCACCGAGTCTCGAAAACTGA
